# Cooperation-Enhanced N–H···π Hydrogen Bonds: Liquid Pyrrole and Its Mixture with Benzene

**DOI:** 10.1021/acs.jpclett.5c04110

**Published:** 2026-02-16

**Authors:** Andrea Sella, Mark Wilson, Miroslava Novoveska, Thomas F. Headen, Adam J. Clancy, Neal T. Skipper, Camilla Di Mino

**Affiliations:** ‡ Department of Chemistry, 4919 University College London (UCL), 20 Gordon Street, London W1CH 0AJ, United Kingdom; § Physical and Theoretical Chemistry Laboratory, Department of Chemistry, 6396 University of Oxford, South Parks Road, Oxford OX1 3QZ, United Kingdom; ∥ The ISIS Neutron and Muon Source (STFC, UK), Rutherford Appleton Laboratory, Fermi Avenue, Harwell Campus, Didcot OX11 0QX, United Kingdom; ⊥ Department of Physics and Astronomy, 4919 University College London (UCL), Gower Street, London WC1E 6BD, United Kingdom

## Abstract

Weak intermolecular interactions are central to the chemical and biological sciences as they dictate the stability, growth, and geometry of larger assemblies. Among weak interactions, NH···π hydrogen bonds are abundant in structural biology, where amines interact with aromatic systems: liquid pyrrole is the ideal test solvent containing both motifs. We therefore combined total neutron scattering and simulation-based refinement to study pure pyrrole and its mixture with benzene. The NH···π interaction between pyrroles is remarkably directional, with NH approaching the center of the ring perpendicularly at 2.11 Å. While the NH···π bond lengths are similar in pyrrole–pyrrole and pyrrole–benzene, the occurrence of the latter is suppressed by a factor of 2. This difference originates from cooperative mechanisms arising from the ability of pyrrole to donate and simultaneously accept a hydrogen bond. Our results clearly show that this traditionally weak interaction can become as short and directional as classical hydrogen bonds.

## Graphical abstract

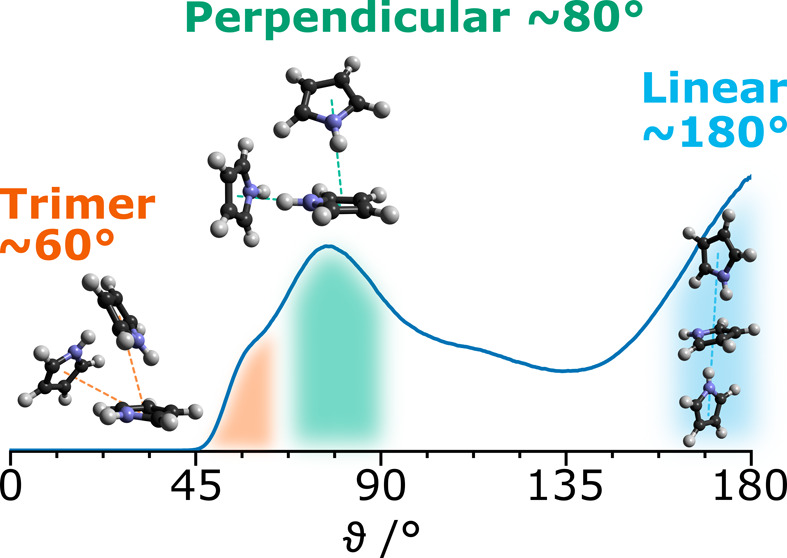

Intermolecular interactions between aromatic rings play a central role in chemistry, materials science, and biology, controlling phenomena from protein folding and drug–receptor binding to charge transport in organic electronics. The molecular arrangement of aromatics in the condensed phase arises from a subtle balance of van der Waals and electrostatic forces and depends critically on the geometry, electronic distribution, and presence of electron-donating or -withdrawing substituents.^1–3^ These groups modulate ring electron density, thereby tuning the molecule’s ability to engage in noncovalent interactions.

Among the simplest heteroaromatics, pyrrole (C_4_H_4_NH) and its derivatives are of particular interest due to their abundance in biological macrostructures (e.g., vitamin B_12_ and heme). In contrast to furans and thiophenes, pyrrole can donate a hydrogen bond through the NH group and possesses a dipole moment that is much larger in magnitude but opposite in direction (0.67 D, 0.53 D, and 1.80 D, respectively), leading to a markedly different balance between π–π, dipole–dipole, and NH···π interactions.^4^

According to the Hunter–Sanders model, aromatic dimers can adopt several preferred arrangements (Figure 1), including so-called parallel-displaced, T-shaped, and the energetically less favorable sandwich geometries, dictated by the competition between π–π repulsion, CH···π attraction, and dispersion.^1,5^ The presence of the nitrogen in the ring defines two extra configurations (head-to-tail and head-to-head) while strongly increasing the likelihood of the T-shaped orientation that is driven by NH···π interactions, at the expense of the Y configuration. The competition between these energetically comparable interactions is reflected in the crystal structure, where pyrrole molecules maximize the overlap between π–π antiparallel displacement and NH···π bonding, pointing the N–H bond toward the midpoint of the C_3_–C_4_ bond opposite the NH site.^6^ The geometry is not perfectly perpendicular as the NH vector intersects the plane of a second pyrrole molecule at ∼70°, leading to antiparallel zigzag chains running along the crystal *z* axis (Figure 2c).

**Figure 1:**
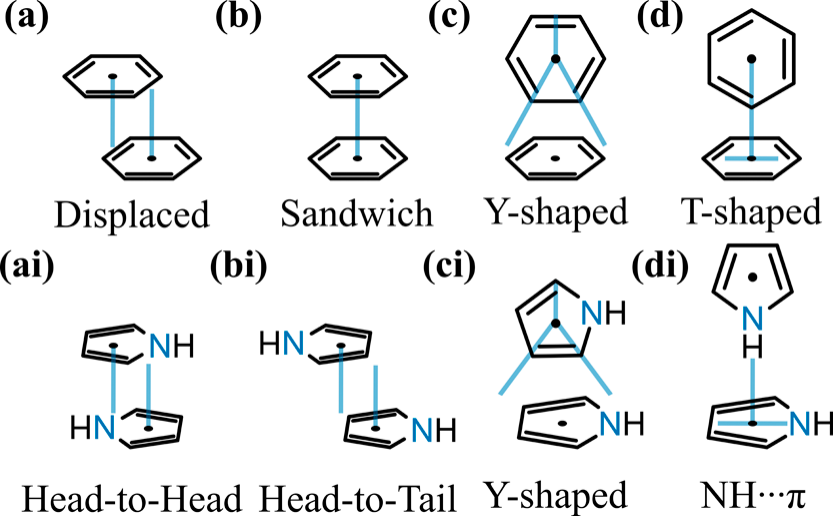
Accessible relative orientations of (a, b, c, and d) benzene and (ai, bi, ci, and di) pyrrole dimers.

**Figure 2:**
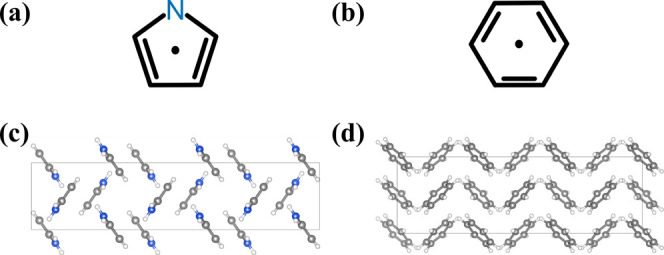
Molecular skeleton of (a) pyrrole and (b) benzene where the Center-of-the-Ring (CoR) is identified by a dot. The 3 × 1 × 1 supercell of crystalline (c) pyrrole and (d) benzene visualized down the [010] axis in VESTA. Note that the faded molecules are at further distances.^7^

In isolated gas phase dimers and trimers, the relative orientation of the two planes decreases to 55°, where π–π interactions are in competition with the weak NH···π interactions.^8^

The length of NH···π and the strength of such interactions have been determined by density functional theory (DFT) calculations, where the distance between H and the center of the aromatic ring (CoR) of two pyrrole molecules was calculated to be 2.3 Å. Interestingly the interaction energies rose from −27.03 kJ mol^–1^ in the dimer to −89.66 kJ mol^–1^ in cyclic trimers, indicating that the presence of additional molecules is energetically more favorable as a consequence of multi-body mechanisms, such as cooperation.^9^

In the liquid state, the room-temperature state of pyrrole, molecules can translate and rotate freely from the geometrical constraints of the solid state. In this more complex system, energetically comparable interactions, such as weak NH···π and CH···π hydrogen bonding, will be highly dynamic, potentially including coexisting perpendicular “T” stacking and parallel displacement (π stacking), with a balance that determines the local and intermediate structure and, consequently, its solvation power and reactivity. However, due to the small system sizes accessible to DFT methods, it remains unclear which molecular geometries maximize cooperativity and how such effects evolve in larger assemblies.

Classical simulations of liquid pyrrole predict the average distance of the NH···π interaction at 2.6 Å, remarkably longer than the average seen in the clusters determined from quantum mechanical methods.^9^ This discrepancy clearly shows that classical modeling alone cannot capture the interdependent mechanisms arising from the concurrent presence of many bonds. Temperature-dependent NIR spectroscopy studies of liquid pyrrole suggest that T-shaped hydrogen bonded aggregates of five or more molecules are the majority species at low temperatures, dissociating into antiparallel stacked pairs as the temperature increases and eventually behaving as independent molecules above 100 °C, but detail is naturally limited by the technique.^10^

To help understand these systems, we have therefore considered pure liquid pyrrole and its mixture with benzene at a 19:1 molecular ratio, to experimentally assess the presence and length of NH···π hydrogen bonds and to identify the complex structuring arising from cooperative mechanisms. We exploited the high sensitivity to hydrogens of total neutron scattering, in combination with extensive hydrogen/deuterium isotopic substitution and data refinement via empirical potential structure refinement (EPSR) simulations, to reveal the ability of pyrrole to simultaneously donate and accept N–H···π hydrogen bonds that constitute the pillars of larger biomolecular assemblies.

Total neutron scattering data on isotopically distinct samples (Supplementary Table 1) were acquired on the NIMROD diffractometer at the ISIS Neutron and Muon Source (Didcot, UK). The wide *Q* range of 0.02–50 Å^–1^ translates into a real space resolution of ∼0.1 Å that is ideal for determining complex liquid behaviors at a molecular level.^11^ The concentration has been carefully selected to ensure that, at this level of dilution, the solvation of benzene is dominated by interactions with pyrrole while still providing a measurable contribution to the experimental neutron scattering signal (Supplementary Figure 2 and Supplementary Note 4). This regime allows us to probe subtle intermolecular interactions, including benzene–pyrrole NH···π hydrogen bonds and aromatic π stacking. Data for the empty instrument and empty null coherent scattering TiZr cells were acquired and subtracted from the data; a 3 mm VNb slab was measured to normalize the data in absolute units (barns atom^–1^ Sr^–1^) via the *GudrunN* routines.^12^ Absorption, inelastic, and multiple scattering events were removed from the total signal using the iterative method developed by Soper.^13^ Simulation-based refinement of the experimental data was performed using the EPSR method via the *Dissolve* software (version 1.8.0; Supplementary Note 2).^14,15^ Structural information about the systems, such as partial distribution functions, *g*_αβ_(*r*), angular radial distribution functions (ARDFs), and spatial distribution functions (SDFs), were extracted within the *Dissolve* GUI and the dlputils routines.^16^ D_5_ pyrrole was synthesized by stirring protio-pyrrole in D_2_O overnight in the presence of 1 equivalent of D_4_ acetic acid.^17^ The mixture was neutralized with potassium carbonate and extracted with dichloromethane. After removal of solvent, the process was repeated twice to give a brown viscous liquid, and distillation under reduced pressure yielded pyrrole D_5_ (86% deuterated). Given the normalization to absolute units of neutron data, we were able to determine residual hydrogenation from the neutron scattering levels and total pair distribution function peak intensities, with this level of hydrogenation taken into account in the data refinement. Partially hydrogenated/deuterated samples were obtained by making a D_5_/H_5_ equimolar mixture to obtain (HD)_5_ pyrrole (Figure 3) before establishing the overall level of deuteration (sample preparation in Supplementary Table 2).

**Figure 3:**
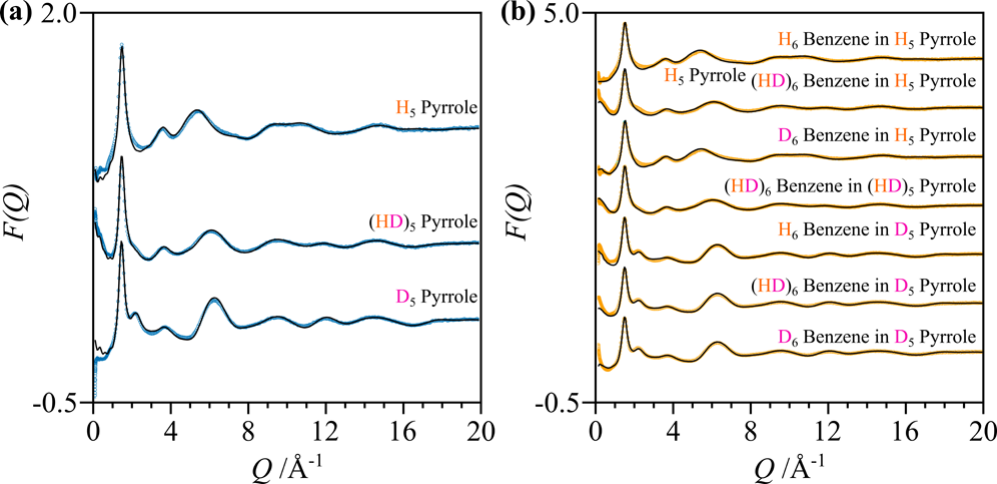
Total neutron scattering data for (a) pure pyrrole (blue circles) and (b) benzene/pyrrole 1:19 molecular mixtures (yellow circles), plotted against the refined functions obtained from *Dissolve* simulations (black line). Note that the total structure factors are normalized to absolute units and plotted here offset along the *Y* axis for clarity.

In a neutron total scattering experiment, under the assumption of single and elastic scattering events, we measure a total structure factor of the form

1 $$F\left(Q\right)=\overset{N}{\underset{\alpha ,\beta \geq 1}{\sum }}\left(2-\delta_{\alpha \beta }\right)c_{\alpha }c_{\beta }b_{\alpha }b_{\beta }S_{\alpha \beta }\left(Q\right)$$

where *α* and *β* are the atomic species, *δ_αβ_* is the Kronecker delta, *N* is the total number of scattering sites, *Q* is the scattering vector defined as $\frac{4\pi }{\lambda }\sin\vartheta$ , *c*_*α*_ and *c*_*β*_ are the atomic number densities, *b*_*α*_ and *b*_*β*_ are the neutron scattering lengths, and *S*_*αβ*_(*Q*) is the Faber–Ziman partial structure factor that contains the structural correlations of each pair of atoms.^18,19^


2 $$S_{\alpha \beta }\left(Q\right)=1+4\pi \rho \int_{0}^{\infty }r^{2}\left(g_{\alpha \beta }\left(r\right)-1\right)\frac{\sin\left(Qr\right)}{Qr}dr$$

The Fourier transformations of *S*_*αβ*_(*Q*) define the site–site partial distribution functions, also referred to as radial distribution functions (RDFs), *g*_*αβ*_(*r*), which are related to the probability density of finding an atom of species α at a distance *r* from an atom of species *β*, for an isotropic system. From this interpretation, it follows that the limit of *g*_*αβ*_(*r*) at long distances is 1.The number of molecules within the solvation shells, identified by the minima of the site–site *g*_*αβ*_(*r*), defines the coordination number *N_αβ_* (*r*_0_) as

$$N_{\alpha \beta }\left(r_{0}\right)=4\pi \int_{r_{\min}}^{r_{\max}}r^{2}g_{\alpha \beta }\left(r\right)\rho_{\beta }dr$$ 3

To investigate the local structure in three dimensions, including relative molecular orientations, we need to define a set of Cartesian axes for each molecule (Figures 4a and 5a), and the definition of ARDFs and SDFs arises naturally from the extension of the definition of RDF in three-dimensional space. The ARDFs are defined as

4 $$g\left(r,\vartheta \right)=\frac{\Delta n\left(r,\vartheta \right)}{\frac{2}{3}\pi \left({\left(r+\Delta r\right)}^{3}-r^{3}\right)\sin\vartheta \Delta \vartheta \rho }$$

where Δ*n*(*r*,*ϑ*) is the number of molecules in distance range *r* + Δ*r* and angle *ϑ* + Δ*ϑ* and *ρ* is the atomic number density. Similarly, the SDFs are a harmonic representation of many-body correlation functions and provide a three-dimensional picture of the arrangement of objects around a central species as a function of their relative orientation.^20^

**Figure 4:**
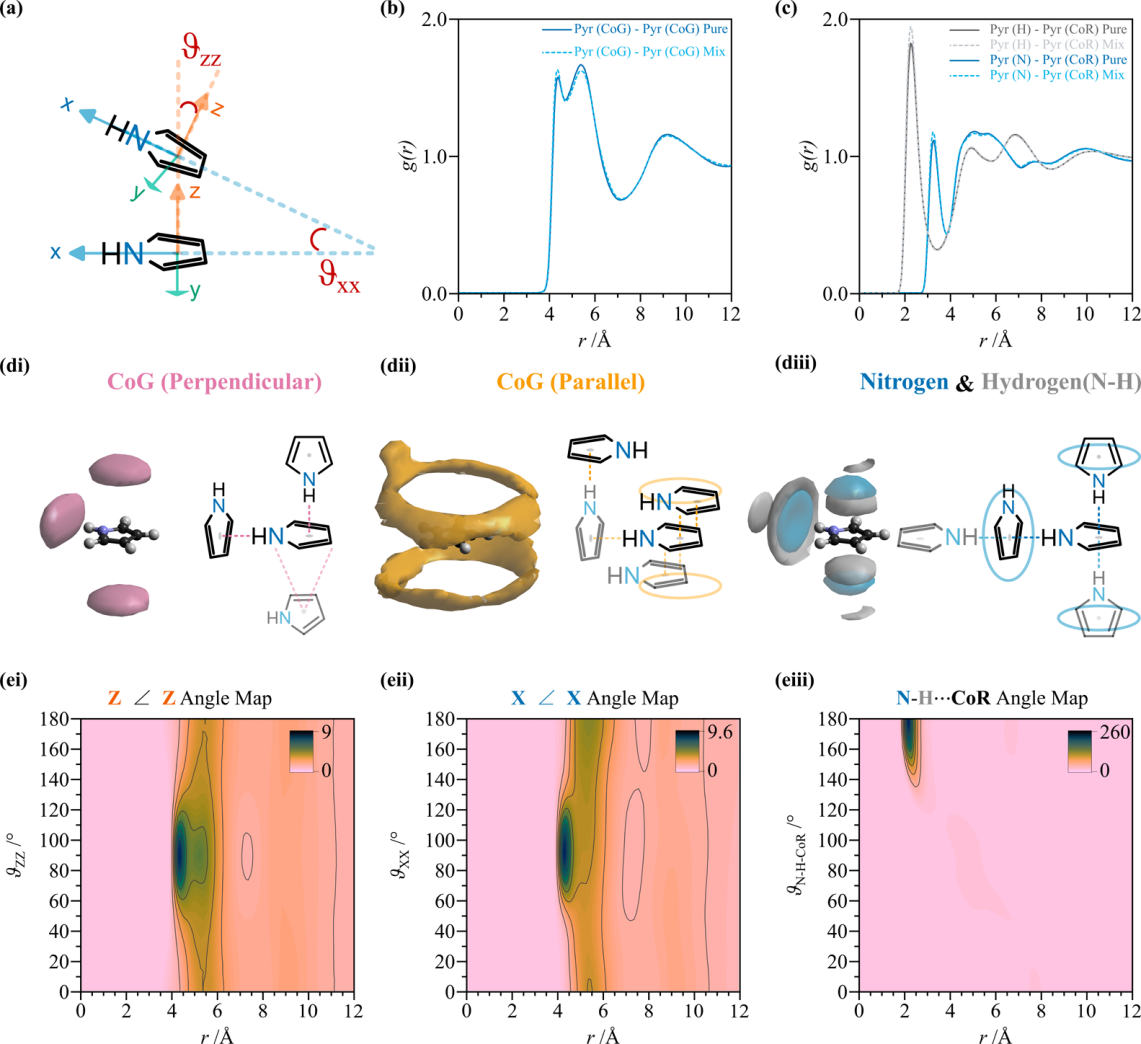
Pyrrole solvation in the pure liquid. (a) Schematic of possible pyrrole–pyrrole relative orientations, where a set of Cartesian axes is defined. (b) CoG–CoG partial radial distribution functions *g_αβ_* (*r*) for pyrrole in the pure liquid (blue solid line) and in the mixture (light blue dashed line). (c) Partial radial distribution functions relative to pyrrole hydrogen (gray) and nitrogen (blue) to pyrrole CoR. (di and dii) Spatial density functions (SDFs) of pyrrole CoG around pyrrole where a second pyrrole molecule is perpendicular (pink) or parallel (yellow) relative to the central pyrrole species within the first solvation shell, <7.5 Å at 20% visualization percentage (see Supplementary Figure 5 for pyrrole in the mix with benzene, pure pyrrole randomly oriented, and different percentages). (diii) Spatial density of pyrrole nitrogen (blue) and hydrogen (gray) within the first solvation shell. Note the templated structure above and below the ring and around the NH group. (ei and eii) Angular radial distribution functions (ARDFs) associated with the orientation of pyrrole out-of-plane axes and pyrrole NH vector. (eiii) N–H···CoR angular map showing remarkable directionality.

**Figure 5:**
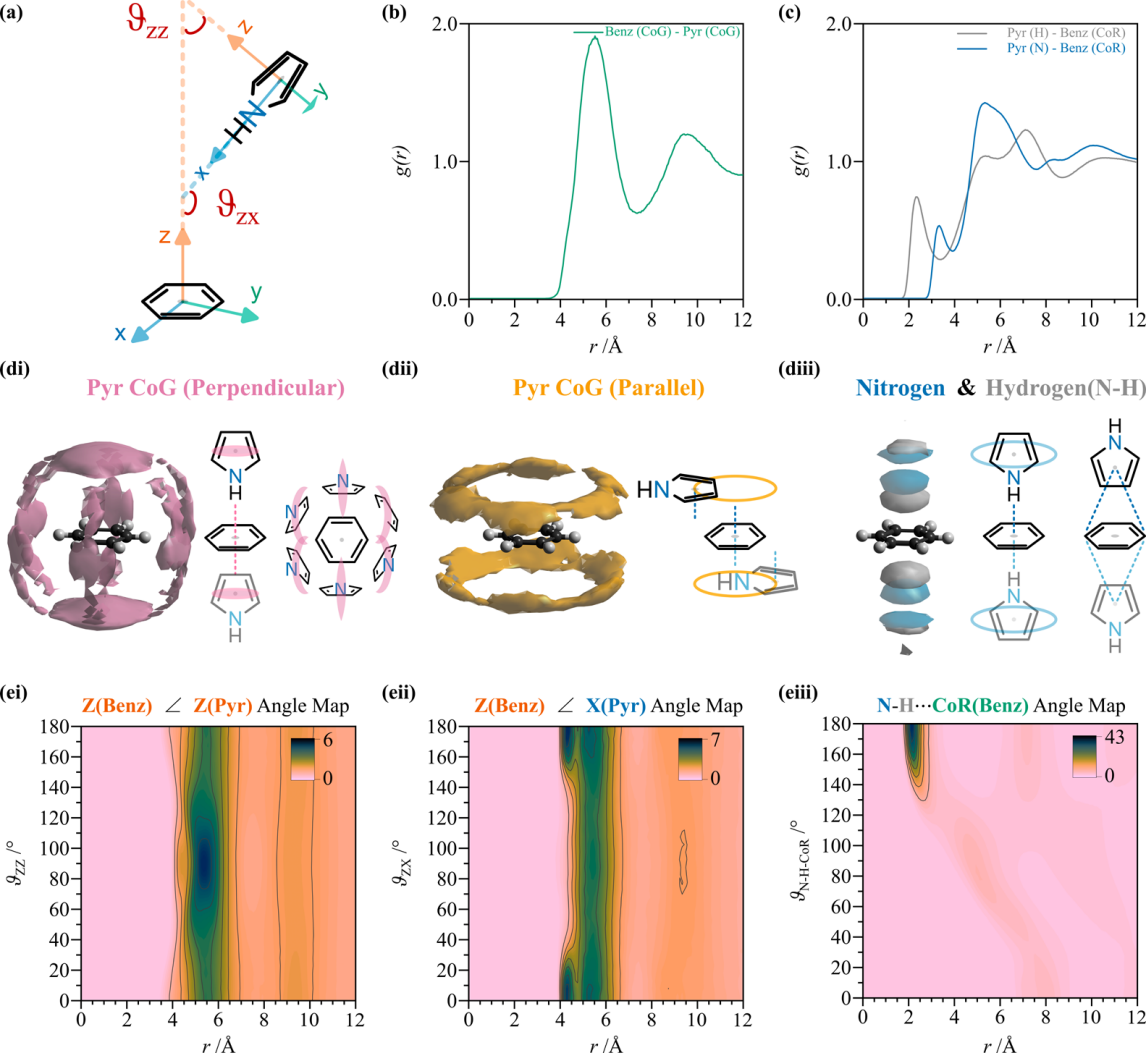
Benzene solvation in pyrrole. (a) Schematic of possible benzene–pyrrole relative orientations. (b) CoG–CoG partial radial distribution functions *g_αβ_* (*r*) for benzene–pyrrole (green solid line). (c) Partial radial distribution functions relative to pyrrole hydrogen (gray) and nitrogen (blue) to benzene CoR. Note the remarkable decrease in intensity from pure liquid pyrrole. (di and dii) Spatial density functions (SDFs) of pyrrole CoG around benzene where a second pyrrole molecule is perpendicular (pink) or parallel (yellow) to the central benzene species within the first solvation shell, <7.5 Å at 20% visualization percentage (see Supplementary Figure 5 for randomly oriented and different percentages). (diii) Spatial density of pyrrole nitrogen (blue) and hydrogen (gray) within the first solvation shell of benzene. Note the templated structure above and below the ring and the inversion at longer distances. (ei and eii) Angular radial distribution functions (ARDFs) associated with the relative orientation of the benzene principal axis and pyrrole out-of-plane axis and pyrrole NH vector. (eiii) NH···CoR angular map showing remarkable directionality.

The intermolecular interactions are modeled by a Lennard-Jones plus Coulomb reference potential of the form

5 $$U_{\alpha \beta }\left(r_{ij}\right)=4\varepsilon_{\alpha \beta }\left[{\left(\frac{\sigma_{\alpha \beta }}{r_{ij}}\right)}^{12}-{\left(\frac{\sigma_{\alpha \beta }}{r_{ij}}\right)}^{6}\right]+\frac{q_{\alpha }q_{\beta }}{4\pi \varepsilon_{0}r_{ij}}$$

where *σ_αβ_* reproduces the atomic core size, while *ε_αβ_* is the depth of the potential well. The site–site interatomic potentials are determined via the Lorentz–Berthelot^21^ mixing rules

$$\sigma_{\alpha \beta }=\frac{\left(\sigma_{\alpha }+\sigma_{\beta }\right)}{2};,\varepsilon_{\alpha \beta }=\sqrt{\varepsilon_{\alpha }\varepsilon_{\beta }}.$$ 6

The input force field and input geometries for pyrrole and benzene were obtained via the LigParGen software, where the atomic charges were sourced from standard OPLS-AA (6-31+G* CHELPG charges for pyrrole and 6-31G* RHF for benzene).^22–24^ In the data-constrained simulations, the NH proton was allowed to exchange. A small addition (in the form of Poisson distribution functions), the empirical potential, is iteratively generated by *Dissolve* from the comparison of the calculated and measured total structure factors and added to the reference potential. The process continues until the best agreement between simulated and measured *F*(*Q*) is reached.

The neutron total scattering data for pyrrole and its mixture at a 1:19 molecular ratio are presented in Figure 3 [(a) blue and (b) yellow circles] alongside the *Dissolve* fits (black line). Excellent agreement is obtained between the simulation and experimental data.

The solvation of pure pyrrole is constituted by two well-defined solvation shells that extend from 4 to 12 Å, as seen from the Center-of-Geometry (CoG)-CoG partial distribution functions *g*_CoG–CoG_(*r*) (Figure 4b). In the first solvation shell (<7.2 Å), pyrrole is surrounded by ∼13 molecules, the same as in pyridine and thiophene, and one extra molecule than that in pure benzene (∼12).^5,25,26^ The first peak of *g*_CoG–CoG_(*r*) is split into two smaller peaks that relate to two different relative orientations. The first peak is associated with a perpendicular arrangement characterized by ∼2 close contacts [*g*_CoG–CoG_(*r*) = 4–5 Å] facilitated by a short T-shaped NH···π interaction (Supplementary Table 4). The second peak (5–6 Å) is broader, and it includes both the parallel and perpendicular arrangements of ∼11 pyrrole molecules. The number of first neighbors is unaffected by the presence of benzene as a minority species (benzene/pyrrole, 1:19), and the local arrangement is largely unperturbed (Figure 4b, solid and dashed lines). We attribute the small change in the relative intensities of the two peaks to a small change in the preferential solvation of pure pyrrole that arises from the excluded volume left behind by benzene. The multidimensional analysis of pyrrole–pyrrole in the mixture is presented in Supplementary Figures 5–7 of the Supporting Information.

A detailed analysis of the orientation of pyrrole molecules in the pure liquid and in the mixture can be achieved by defining a set of Cartesian axes originating from the center-of-geometry (CoG) of both pyrrole and benzene. We define the *x* axis in pyrrole as the NH direction (i.e., the direction of the molecular dipole), while for benzene, the *x* axis is chosen along a C–H bond, with the *z* axis set perpendicular to the ring plane of the molecules (Figures 4b and 5b). In this manner, we can plot the full and conditional spatial density functions (SDFs) that represent the probability of finding a second pyrrole molecule within a certain distance and orientation.

We first consider the spatial density relative to the molecules within the pyrrole’s first solvation shell (<7.5 Å). At low visualization percentages, corresponding to the most likely ≤10% of molecules in the first solvation shell, the lobes are localized above and below the ring and directly in proximity of the NH group (Supplementary Note 2 and Supplementary Figure 5a). However, at higher percentages (>10%), the SDFs cannot be clearly related to the aromatic arrangements presented in Figure 1 (Supplementary Figure 7). By defining the angles *ϑ_zz_* and *ϑ_xx_* between the *z* and *x* axes of two pyrrole molecules, we can distinguish between those pyrrole molecules that are perpendicular (*ϑ*_*zz*_ = 90 ± 10°; Figure 4di) or parallel (*ϑ*_*zz*_ = 0 ± 10°; Figure 4dii) to the pyrrole molecular plane and plot the conditional SDFs.

The perpendicular arrangement is characterized by three lobes already present in the full SDFs, highly localized in the area above and below the aromatic ring, and alongside the direction of the NH group. In the ARDFs (Figure 4ei and eii) that represent the probability density of finding a molecule at a distance *r* and angle *ϑ* relative to a central species, we see a first intense peaks at 90° and a less intense 90° peak at slightly longer distances (Figure 4ei). The height of the first peak reaches an intensity of 9. For reference, in pure benzene, this peak has an intensity of 2.4 (Supplementary Figure 3eii). Similarly for pyridine and thiophene, where dipole–dipole interactions are introduced, the peak intensity remains lower than 3.^25,26^ The latter observation is a clear indication of a preferential perpendicular arrangement between pyrroles, enforced by NH···π hydrogen bonding.

To unambiguously identify these perpendicular pyrrole molecules as interacting via NH···π, we can plot *g*_CoR–H_(*r*) and *g*_CoR–N_(*r*) (Figure 4c). The first intense peak in *g*_CoR–H_(*r*) is at 2.29 Å, while the first peak in *g*_CoR–N_(*r*), equally as sharp as for *g*_CoR–H_(*r*), is found at 3.25 Å. These two peaks are at ∼1 Å apart, coincident with the intramolecular NH distance, presenting a first strong indication of the high directionality of the NH···π interaction. A further confirmation of the remarkable directionality is the intense peak at 180° in the N–H···CoR angle map in Figure 4eiii. The maximum is reached at 180° at a distance of 2.11 Å. This distance is among the shortest reported XH···π interactions in the liquid state and is comparable in length and directionality with classical hydrogen bonds (X = O, N, and C; OH···O = 1.85 Å in water; and NH···N = 2.25 Å in liquid ammonia).^27,28^

The directionality of the NH···π bond inferred from the average distances between the CoR and NH group (Figure 4b) is further confirmed by the highly localized spatial distribution of hydrogen and nitrogen around the pyrrole CoR and NH group (Figure 4diii). Interestingly, the average location of pyrrole H around the NH group is a ring, showing the free rotation of a pyrrole ring around its CoR with respect to its *z* axis. Moreover, the pyrrole hydrogen spatial density presents with a second lobe that lies in the direction of the NH group but at longer distances than the nitrogen-related density.

The ARDFs of Figure 4ei show that the likelihood of parallel relative geometry of two pyrrole molecules is substantially lower than that for perpendicular contact. However, the displaced geometry can shed light on the complementary arrangement within the first solvation shell. If we now look at those pyrrole molecules lying parallel to the central species (Figure 4dii), we see that they form a halo above and below the molecule but displaced from the *z* axis to minimize electrostatic repulsion, as expected for small aromatics.^1–3^ Interestingly, a lobe appears within the first solvation shell directly above (and below by symmetry) the NH group at greater distances from the halo of displaced aromatics. It is worth noting that a second lobe appears in the direction of the NH vector in the SDF of Figure 4dii when the integration limit is extended to a longer distance (Supplementary Figure 7c).

These lobes at longer distances indicate the consistent average presence of additional pyrrole molecules lying antiparallel (i.e., head to head; Figure 1ai) to the central species, which accepts an NH···π hydrogen bond from a molecule that is already hydrogen bonded to the central species (Supplementary Figure 8d). Thus, when NH···π is formed, a third molecule approaches, accepting a hydrogen bond from the one before and so on in a cooperative manner. The CoR–H coordination number is 1, as an indication that aminic hydrogen is on average always involved in NH···π bonds (Supplementary Table 4). This mechanism is facilitated by the ability of the faces of the aromatic rings to act independently; i.e., the formation of one bond does not affect the ability of forming a second one to the opposite face. In pyrrole, the probability of having one face involved in one NH···π interaction is 52%, while the probability of having two hydrogen bonded molecules to opposite faces is 23% (Supplementary Table 4). This highly structured solvation behavior is dominated by cooperative cyclic motifs initiated by strong NH···π, as previously seen for OH···π, and is also reminiscent of what has been observed in the gas phase pyrrole trimers, a motif preserved in the liquid (Supplementary Figure 11).^9,29^

In order to isolate the NH···π interaction, we can disentangle the behaviors of pyrrole as a hydrogen bond donor and acceptor by introducing a different π system. Benzene provides an ideal test molecule, where the delocalized π system can solely accept hydrogen bonds, hindering the formation of the cooperative cyclic motifs. In addition, benzene’s high symmetry and absence of electron-donating heteroatoms and substituents will impede dipole–dipole interactions, which would otherwise compete with the NH···π_benzene_ interaction of interest.

To explore how the introduction of benzene affects the structure of pyrrole, we can plot the RDFs, ARDFs, and SDFs of pyrrole around benzene (Figure 5). The molecular volume of benzene is slightly larger than that of pyrrole (147 vs 115 Å^3^ molecule^–1^), and this will be reflected in the *g*_CoG–CoG_(*r*) distances. The splitting of the first peak as seen for pure pyrrole disappears in benzene–pyrrole *g*_CoG–CoG_(*r*) (Figure 5b) leaving behind a shoulder at 4.3 Å and a second, broad peak at 5.5 Å that is more intense than the second peak of the pyrrole–pyrrole interaction (Figure 4b and Supplementary Figure 3a). These changes suggest both a decrease in the number of pyrrole molecules interacting closely and perpendicular to the benzene and a change in the preferential orientation of pyrrole molecules in the first solvation shell of benzene. The most likely location of pyrrole molecules that are perpendicular to the benzene plane is above and below the ring (Figure 5di). These arrange either in T-shaped (NH···π) or Y-shaped configurations (Figures 1ci and di), as for pure pyrrole. Around the ring, the solvation is dominated by pyrrole molecules located preferentially between two benzene CH groups in a bifurcated fashion, characteristic of aromatic Y stacking. Similarly to pure pyrrole, the pyrrole rings that are parallel to a central benzene are located in a halo with a displaced arrangement above and below benzene (Figure 5dii).

The *g*_CoR–H_(*r*) presents a peak at 2.35 Å, for hydrogen bonded NH, slightly longer and markedly less intense than in the pyrrole–pyrrole case (Figure 5c and Supplementary Figure 3b). At the same time, the first peak in *g*_CoR–N_(*r*) appears at 3.3 Å and is broader, consistent with a highly directional NH···π interaction. The angle map of N–H···CoR_(Bz)_ shows a peak at 180°, less intense than that seen for pure pyrrole (Figure 5eiii). The NH···π interaction is therefore present in the pyrrole–benzene system but weaker than that in pure pyrrole due to the absence of the cyclic cooperative mechanisms arising from the ability of pyrrole to simultaneously donate and accept hydrogen bonds. The occurrence of the NH···π interaction is remarkably less frequent: we find that the average CoR–H coordination number within a sphere of 3.5 Å radius is 0.54 (∼1.0 in pure pyrrole). It is worth highlighting that the occurrence of NH···π in benzene pyrrole is very similar to the OH···π contacts in benzene methanol, with probabilities of 0.53, 0.40, and 0.07 of having 0, 1, or 2 contacts (Supplementary Table 4).^29^ The faces of the benzene ring here are acting statistically independently, as in the benzene methanol case.

The spatial arrangement of H and N around a benzene molecule is above and below the ring, as in the case for pure pyrrole (Figure 5diii). However, within 7.5 Å, an inverted spatial arrangement is present at further distances, where N is closer to the CoR than H, suggesting aromatic Y stacking. The pyrrole–benzene Y average configuration is confirmed by the ARDFs of Figure 5ei and eii, which present two consecutive peaks at both 0° and 180° in the case of the *x*_pyr_–*z*_benz_ axis orientation and two consecutive peaks at 90° for *z*_pyr_–*z*_benz_, of which the second is more intense. Compared to pure pyrrole, the intensity of the second peak at longer distances is much reduced but still present. The Y-stacking configuration in pure pyrrole is the result of local packing, where a pyrrole molecule binds to a π ring of a pyrrole that is already acting as a H donor to a separate molecule. In the benzene pyrrole mixture, this configuration is highly favorable as the H of C_3_/C_4_ furthest from N is the most positive region of the molecule (after NH) and additionally because the Y shape exposes the NH group, allowing it to participate in cyclic pyrrole–pyrrole cooperative interactions. Notably, pyrrole’s self-solvation in the presence of benzene is preserved (Supplementary Figures 3–5). The presence of the Y benzene–pyrrole configuration explains the small difference in intensity in pyrrole–pyrrole *g*_CoG–CoG_(*r*), which indicates moderately enhanced pyrrole–pyrrole interactions in the presence of benzene.

In summary, neutron total scattering augmented with H/D isotopic substitution and simulation-based refinement allowed us to explore the liquid structure of pure pyrrole and its mixture with benzene, revealing a strongly structured network of cooperative cyclic NH···π interactions. Contrarily to what is seen in the solid and gas phases, in the liquid state, the NH group of pyrrole lies perpendicular to the aromatic ring of a second molecule, with pyrrole's aminic hydrogen approaching the ring at distances as close as 2.11 Å, with an average of 1 hydrogen bonding interaction per NH group. These NH···π interactions are comparable in length to the more common OH···π bonds and even overlap with classical hydrogen bonding distances (NH···O and NH···N). Remarkably, the presence of benzene leaves pyrrole–pyrrole interactions unaffected, while pyrrolic NH···π interactions form to benzene at similar distances but at lower probability owing to the absence of the cooperativity seen in pure pyrrole, arising from its simultaneous nature as a hydrogen bonding donor and acceptor. Together, these results show that the strength of specific NH···π intermolecular bonds lies in their molecular environments and that the otherwise very weak XH···π interactions may be enhanced by cooperative mechanisms.

## Supplementary Material





## Data Availability

Unprocessed neutron data are available at 10.5286/ISIS.E.RB2510229-2.

## Notes

Neutron scattering theory (Supplementary Note 1), EPSR theory (Supplementary Note 2), additional structural data (Supplementary Note 3), neutron scattering weights (Supplementary Note 4) (PDF)
Transparent Peer Review report available (PDF)
