# Supplementary material for: Cooperation-Enhanced N–H···π Hydrogen Bonds: Liquid Pyrrole and Its Mixture with Benzene
Source: J Phys Chem Lett. 2026 Feb 16;17(8):2264–70. doi: 10.1021/acs.jpclett.5c04110 (PMC12951553; doi:10.1021/acs.jpclett.5c04110)
Supplement: Supplementary file 1 [file jz5c04110_si_001.pdf]

Supplementary Information for "Cooperation Enhanced N-H···π Hydrogen Bonds: Liquid Pyrrole and its Mixture with Benzene"

Andrea Sella,[1†] Mark Wilson,[2†] Miroslava Novoveska,[1] Thomas F. Headen,[3] Adam J. Clancy,[1] Neal T. Skipper,[5,*] Camilla Di Mino[2,*]

1) Department of Chemistry, UCL, 20 Gordon Street, London, W1CH 0AJ, UK
2) Physical and Theoretical Chemistry Laboratory, Department of Chemistry, University of Oxford, South Parks Road, OX1 3QZ, UK
3) ISIS Neutron and Muon Source, Rutherford Appleton Laboratory, Fermi Avenue, Harwell Campus, Didcot, OX11 0QX, UK
4) Department of Physics and Astronomy, UCL, Gower Street, London, WC1E 6BD, UK
†These authors contributed equally.
E-mails: camilla.dimino@chem.ox.ac.uk; n.skipper@ucl.ac.uk

Supplementary Note 1: Neutron Scattering Theory

In a total neutron scattering experiment we measure a total structure factor, *F(Q),* that after correction for multiple and inelastic scattering events is:

$$F(Q) = \sum_{\alpha,\beta\geq 1}^{N}(2-\delta_{\alpha\beta})c_\alpha c_\beta b_\alpha b_\beta S_{\alpha\beta}(Q)\ , \tag{S1}$$

where $Q = \frac{2\pi}{\lambda}\ sin\vartheta$ is the momentum transfer vector; $\delta_{\alpha\beta}$ is the Kronecker delta; c_α, c_β are the atomic concentrations of species α and β; b_α, b_β are the neutron scattering lengths; and $S_{\alpha\beta}(Q)$ are the Faber-Ziman partial structure factors that contain the site-site correlation of each atom species.[1]

From the Fourier Transformation of the total structure factors, we define the total pair distribution functions, *G(r),* as

$$G(r) = \frac{1}{(2\pi)^3\rho_0}\int_0^\infty 4\pi Q^2 F(Q)\frac{\sin(Qr)}{Qr}\ dQ\ , \tag{S2}$$

where ρ_0 is the atomic number density; they are linked to the partial radial distribution functions of equation (2) of the main text via the sum

$$G(r) = \sum_{\alpha,\beta\geq 1}^{N}(2-\delta_{\alpha\beta})\ c_\alpha c_\beta b_\alpha b_\beta(g_{\alpha\beta}(r)-1)\ . \tag{S3}$$

Supplementary Note 2: EPSR Theory

Total neutron scattering data were interpreted via the Empirical Potential Structure Refinement (EPSR) method within *Dissolve (v1.8.0).* By constraining the Monte Carlo simulation with the neutron data, we obtained a molecular ensemble that represents the real system, bypassing the challenges of data inversion from reciprocal to real space. For a system of *i* distinct atomic sites, the number of total correlations is

$$N = \frac{i(i+1)}{2}\ . \tag{S4}$$

It is clear that the inversion methodology is not possible in systems with a number of degrees of freedom that is higher than the number of independent isotopic contrasts. Even for the simplest systems (e.g., water), the inversion method would not provide the amount of structural information we can extract from the EPSR (e.g., three-dimensional analysis).

The simulation is set up from physiochemical knowledge of the system in terms of molecular geometries (Supplementary Table 4), liquid densities, temperature, and interatomic potentials (Supplementary Table 3). The method equilibrates the molecular ensemble for ~2000 steps, after which the total free energy reaches a plateau. When the empirical potential of amplitude 7 is switched on, the method updates the intermolecular potential every 5 steps by adding a small contribution to the total to improve the agreement with the experimental data. The procedure continues until the best agreement (lowest R-factor) is reached. The simulation subsequently accumulates >100,000 configurations from which we extract the average local and intermediate-range structure of the system. The high number of configurations was essential to accurately capture benzene-benzene contacts in solutions at this concentration, being benzene the minority species.

Pure liquid pyrrole was modelled in a cubic simulation box of side 48.66 Å containing 1000 molecules to reproduce the experimental density of 0.966 $g{\cdot}cm^{-3}$ (0.0868 $atoms{\cdot}Å^{-3}$). Benzene-pyrrole 1:19 molecular mixture was modelled in a cubic simulation box of side 83.63 Å containing 250 molecules of benzene and 4750 of pyrrole at a density of 0.960 $g{\cdot}cm^{-3}$ (0.0863 $atoms{\cdot}Å^{-3}$). The density of the mixture was calculated from a weighted average of pyrrole and benzene densities with the relative molecular abundances that reflected accurately the experimental densities measured via an Anton Paar Density Meter DMA 4100M (0.966±0.001 $g{\cdot}cm^{-3}$ for pure pyrrole and 0.960±0.001 $g{\cdot}cm^{-3}$ for pyrrole benzene). From the refined molecular ensemble, we extracted the structural information (RDF, ARDFs, SDFs) using the *Dissolve* GUI and the *dlputils* routines.[2, 3]

The Spatial Density Functions (SDFs) are a harmonic representation of many-body correlation functions and allowing to picture in three dimensions the most probable location of a molecular species around another, as a function of distance and orientation.[3-5] A set of axes needs to be defined for each molecular species (Figure 4a, 5a). The resulting data can be visualised as a function of the molecular fractions by filling the space from the most probable to the least likely location. For example, a lower percentage (5-15%) can be used to focus on specific bonding motifs, while a higher percentage (30-50%) is useful to compare trends in solvation behaviours. These percentage limits are highly dependent on the systems of study. It is worth pointing out that for establishing comparisons between the solvation of different central molecules by the same species, or the same central molecule by two different species, the visualisation percentage and distance cut-off need to be set at the same level and distance.

Supplementary Note 3: Additional Structural Data

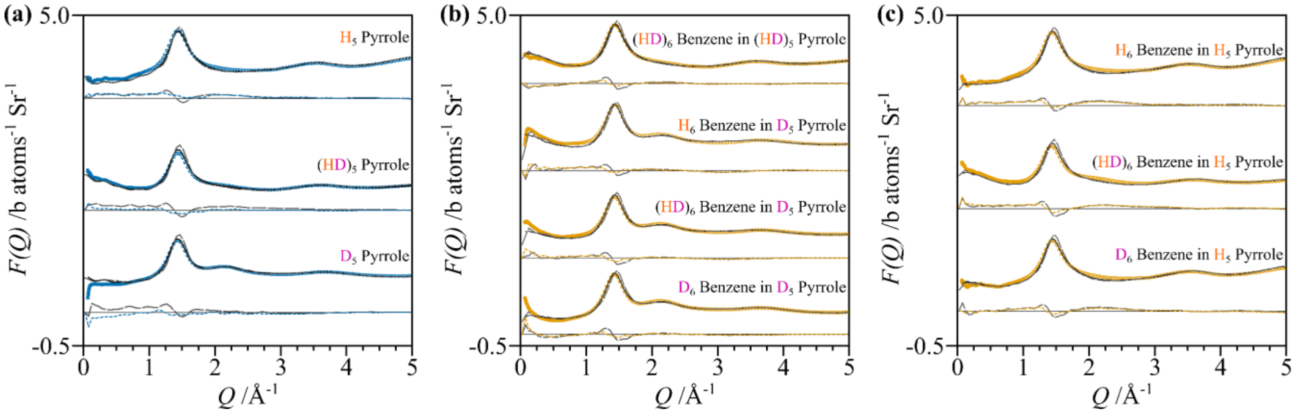

Supplementary Figure 1 *Total structure factors F(Q)s of Figure 3 plotted between 0-5 $Å^{-1}$ to highlight structural refinement against the model with and without Empirical Potential. Data (blue, yellow circles), Dissolve modelled without refinement (grey, long dash), EPSR refined (black, solid), and fit residuals.*

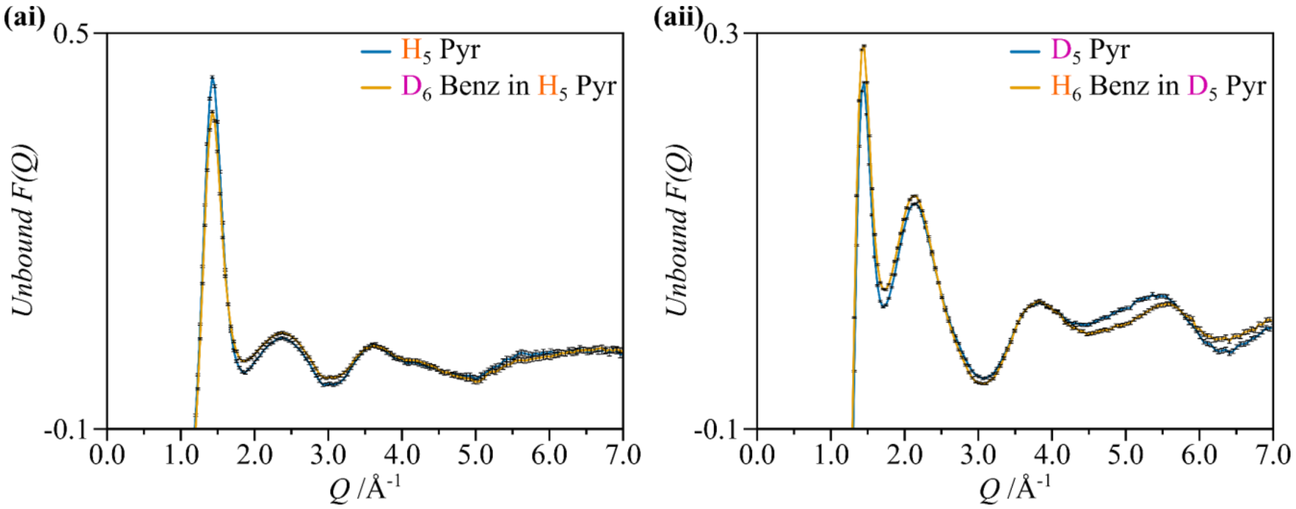

Supplementary Figure 2 *Unbound F(Q)s obtained by subtracting the bound F(Q)s produced by Dissolve from the experimental neutron data, plotted with the relative experimental uncertainties. The contribution of the intermolecular structure is dominant in the range 0.1 – 3.0 $Å^{-1}$. The scattering intensities vary in a trend consistent with the scattering length densities of H and D samples.*

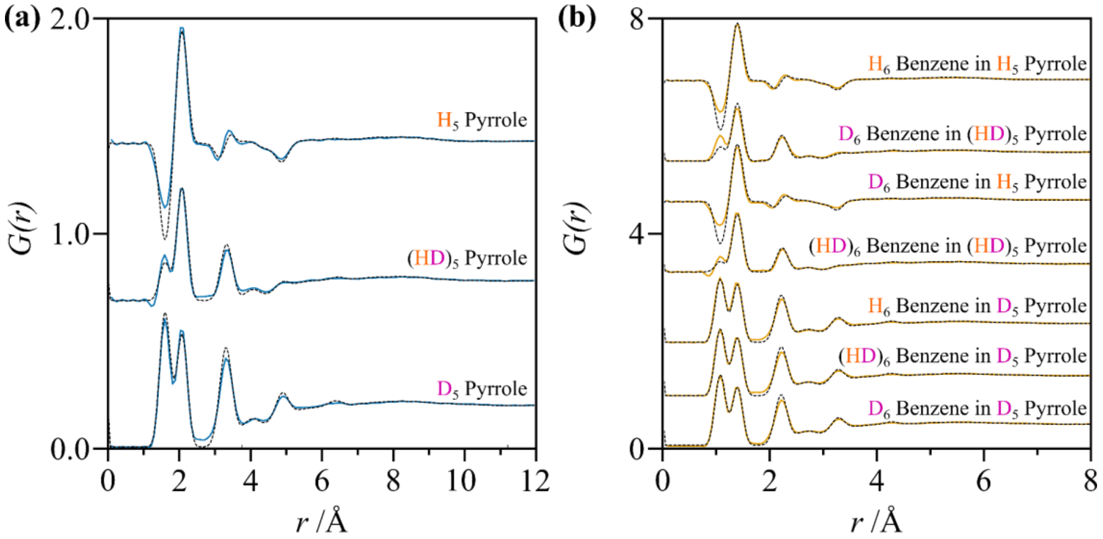

Supplementary Figure 3*. Total pair distribution functions G(r) obtained from direct Fourier Transformation of the total structure factors F(Q) (Lorch broadening function, Q_{max} 50Å^{-1}) for* ***(a)*** *pyrrole (blue, solid line) and* ***(b)*** *benzene-pyrrole (yellow solid line) plotted against the model (black dashed line).*

Supplementary Table 1. *List of isotopically distinct samples for pyrrole and benzene-pyrrole at 1:19 molecular ratio*

Pure Pyrrole	Benzene in Pyrrole 1:19
D_5 pyrrole	D_6 benzene in D_5 pyrrole
H_5/D_5 pyrrole	H_6 benzene in D_5 pyrrole
H_5 pyrrole	$(HD)_6$ benzene in D_5 pyrrole
	D_6 benzene in H_5 pyrrole
	D_6 benzene in $(HD)_5$ pyrrole
	$(HD)_6$ benzene in $(HD)_5$ pyrrole
	H_6 benzene in H_5 pyrrole

Supplementary Table 2. *Experimental weights and molecular ratios used to produce the isotopically distinct samples.*

Pyrrole Isotopologue	Benzene Isotopologue	wt. Benzene /g	wt. Pyrrole /g	Molar Ratio
D_5	D_6	0.085	1.388	19.051
D_5 / H_5	D_6	0.069	1.088	19.000
H_5	D_6	0.087	1.312	18.907
D_5	H_6	0.063	1.055	18.142
D_5 / H_5	D_6/ H_6	0.080	1.315	19.205
H_5	H_6	0.081	1.325	19.044

Supplementary Table 3. *Lennard-Jones parameters and charges for pyrrole and benzene obtained from [6, 7].*

Species	σ /Å	Ɛ /kJ mol^{-1}	q /e
H(-N)	0.0	0.0	0.317
N	3.25	0.71128	-0.239
C(-N)	3.55	0.29288	-0.163
H(-C-N)	2.42	0.12552	0.155
C (-C)	3.55	0.29288	-0.149
H (-C-C)	2.42	0.12552	0.118
C_{benz}	3.55	0.29288	-0.115
H_{benz}	2.42	0.12552	0.115

Supplementary Table 4. *Intra-molecular structural parameters (bond lengths and angles) for pyrrole and benzene.*

	Bond Length /Å		Bond Angle /°
C(-N)-C(-C)	1.37	N-C-C	108.0
C(-C)-C(-C)	1.42	C-C-C	107.3
-C(-C) - **H (-C-C)**	1.08	H(-N)-N-C	125.3
**H(-N)-N**	1.01	N-C-H(-C-N)	120.8
C(-N)- **H(-C-N)**	1.08	H(C)-C-C	131.2
		C(-N)-C-H(-C-C)	125.8
		H(-C-C)-C(-C)-C(-C)	126.9
C_{benz}-C_{benz}	1.40	C-C-C	120.0
H_{benz}-C_{benz}	1.08	C-C-H	120.0
		H-C-H	120.0

Supplementary Table 5. *Coordination numbers for pyrrole and benzene CoG-CoG, and for pyrrole-benzene CoR and N, H pyrrole atom sites. The integration limit is selected as the minimum of the first $g_{\alpha\beta}(r)$ peak. Note that for CoG-CoG coordination number, the breakdown into probabilities of contacts exceeds 4 and therefore it has been omitted.*

**Benzene - Benzene**	**Integration Limit /Å**	**Coordination Number**	**0 Contacts /%**	**1 Contact /%**	**2 Contacts /%**	**3 Contacts /%**	**4 Contacts /%**
CoG-CoG	4.7	0.02	98	2	0	0	0
	7.2	0.70	46	36	14	4	0
	7.5	0.75	43	37	15	5	0
**Benzene-Pyrrole**	**Integration Limit /Å**	**Coordination Number**	**0 Contacts /%**	**1 Contact /%**	**2 Contacts /%**	**3 Contacts /%**	**4 Contacts /%**
CoG-CoG	4.7	0.7	43	43	13	1	0
	7.2	12.3	-	-	-	-	-
	7.5	13.4	-	-	-	-	-
CoR-H	3.5	0.54	53	40	7	0	0
H-CoR	3.5	0.54	97	3	0	0	0
CoR-N	4.5	0.92	34	45	18	3	0
N-CoR	4.5	0.92	95	5	0	0	0
**Pyrrole-Pyrrole (Pure)**	**Integration Limit /Å**	**Coordination Number**	**0 Contacts /%**	**1 Contact /%**	**2 Contacts /%**	**3 Contacts /%**	**4 Contacts /%**
CoG-CoG	4.7	1.9	6	28	41	21	4
	7.2	12.9	-	-	-	-	-
	7.5	14.0	-	-	-	-	-
CoR-H	3.5	1.0	25	52	23	1	-
H-CoR	3.5	1.0	6	88	6	-	-
CoR-N	4.5	1.8	9	31	37	19	4
N-CoR	4.5	1.8	1	37	45	16	1
**Pyrrole-Pyrrole (Mix)**	**Integration Limit /Å**	**Coordination Number**	**0 Contacts /%**	**1 Contact /%**	**2 Contacts /%**	**3 Contacts /%**	**4 Contacts /%**
CoG-CoG	4.7	1.8(5)	7	29	41	20	3
	7.2	12.1	-	-	-	-	-
	7.5	13.3	-	-	-	-	-
CoR-H	3.5	0.96	26	53	20	1	0
H-CoR	3.5	0.96	9	86	5	0	0
CoR-N	4.5	1.72	10	33	37	17	3
N-CoR	4.5	1.72	2	40	43	14	1

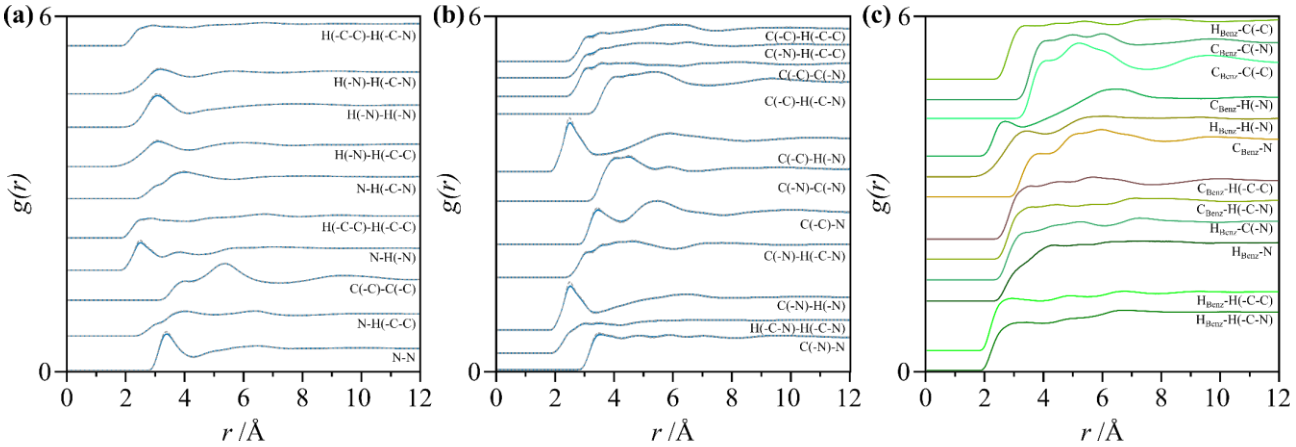

Supplementary Figure 4. *Site-Site partial radial distribution functions* $g_{\alpha\beta}(r)$ *for* ***(a,b)*** *pure pyrrole with (grey, dashed line), and without (blue, solid line) benzene and* ***(c)*** *benzene-pyrrole at 1:19 molecular ratio.*

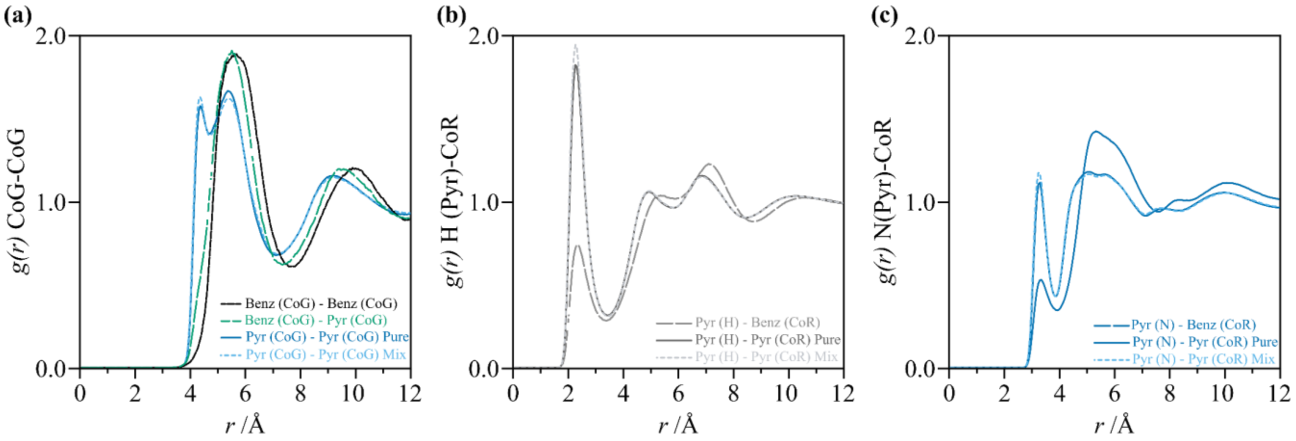

Supplementary Figure 5. *Centre-of-Geometry (CoG) - CoG partial radial distribution functions of Main Text Figure 4 and 5 plotted on the same graph for direct comparison.*

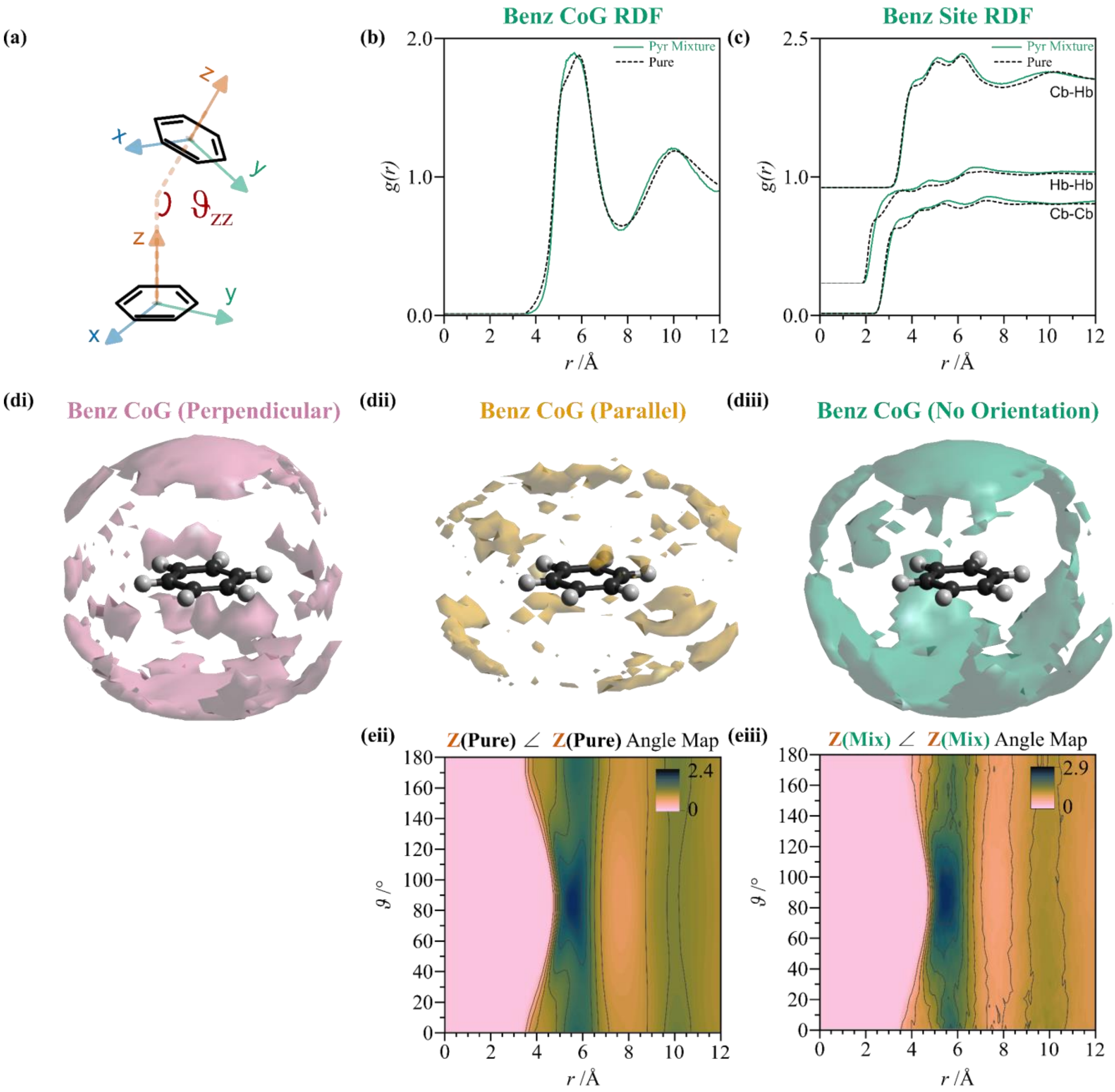

Supplementary Figure 6*. Benzene-benzene solvation in benzene-pyrrole mixtures at 1:19 molecular ratio.* ***(a)*** *Schematic of benzene-benzene orientations;* ***(b)*** *CoG-CoG partial radial distribution functions, g(r), for benzene in the pure liquid (black dashed line) and in the mixture (green solid line);* ***(c)*** *atom-atom partial radial distribution functions, g(r), for benzene in the pure liquid (black dashed line) and in the mixture (green solid line);* ***(di,dii,diii)*** *Spatial density function, SDFs, of benzene CoG around benzene in the mixture with pyrrole where the benzene molecules are perpendicular (pink), parallel (yellow), and randomly orientated (green) relative to the central benzene species.* ***(eii, eiii)*** *Angular Radial Distribution Functions ARDFs associated with the relative orientation of benzene principal axis z in the pure liquid and in the mixture with pyrrole. Data for pure benzene reproduced from Headen [8].*

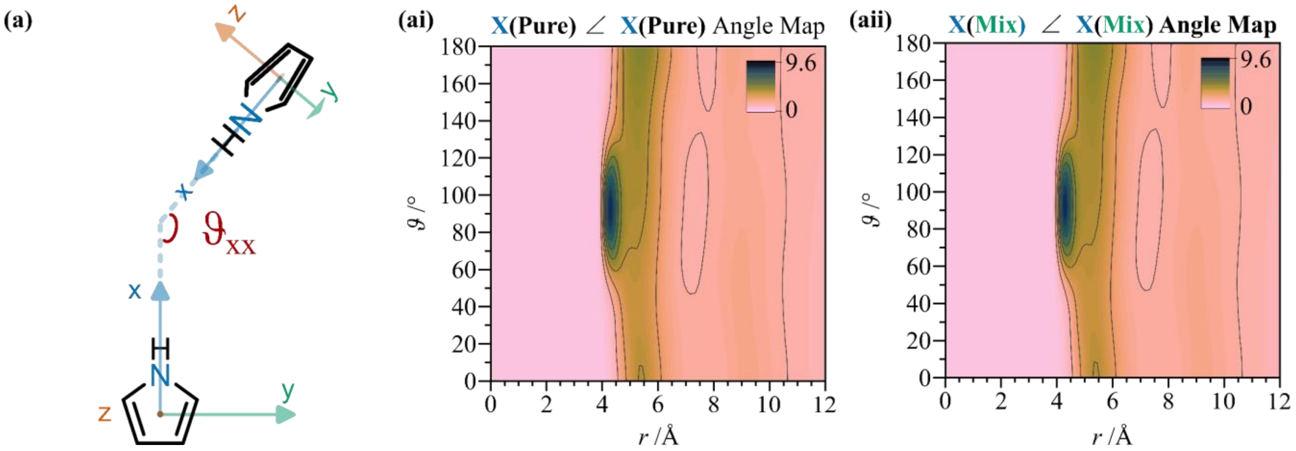

Supplementary Figure 7. *Angular Radial Distribution Functions ARDFs associated with the relative orientation of NH vector in* ***(ai)*** *pure liquid pyrrole and* ***(aii)*** *in the mixture with benzene.*

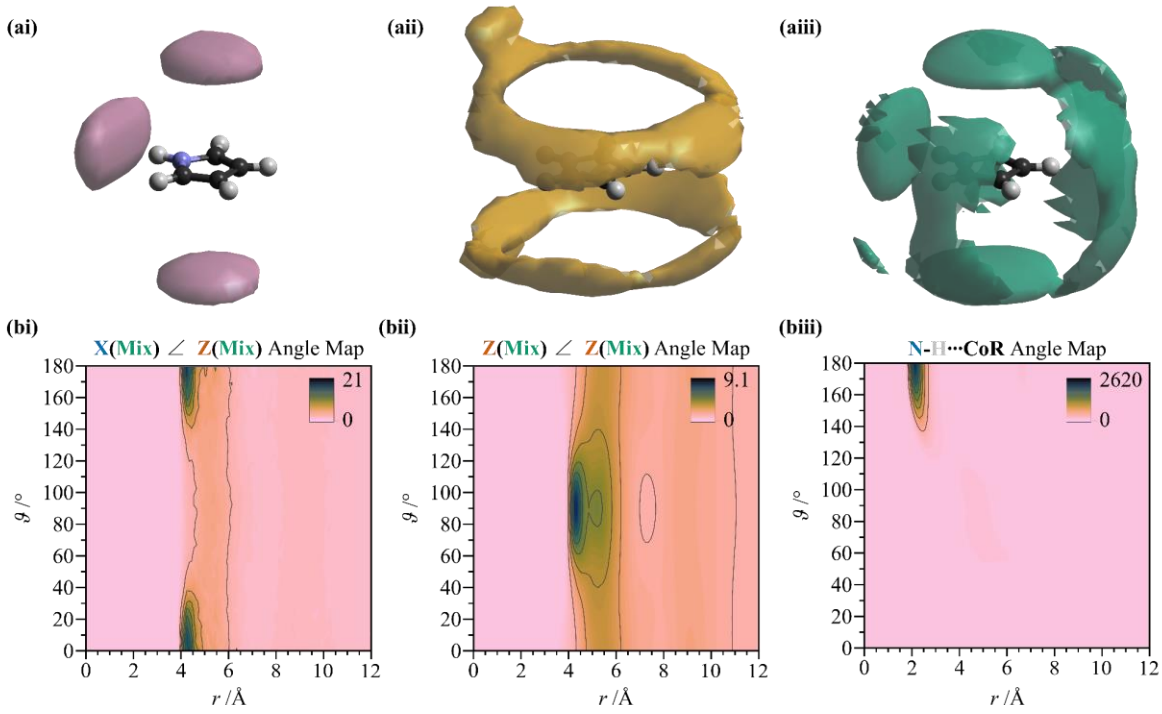

Supplementary Figure 8. (ai,aii,aiii) *Spatial density function SDFs of pyrrole CoG around pyrrole in the mixture with benzene where the pyrrole molecules are perpendicular (pink), parallel (yellow), and randomly orientated (green) relative to the central pyrrole species;* ***(bii, biii)*** *Angular Radial Distribution Functions ARDFs associated with the relative orientation of pyrrole NH vector in the mixture with benzene;* ***(biii)*** *N-H···CoR(pyr) angle map between two pyrrole molecules in the mixture with benzene.*

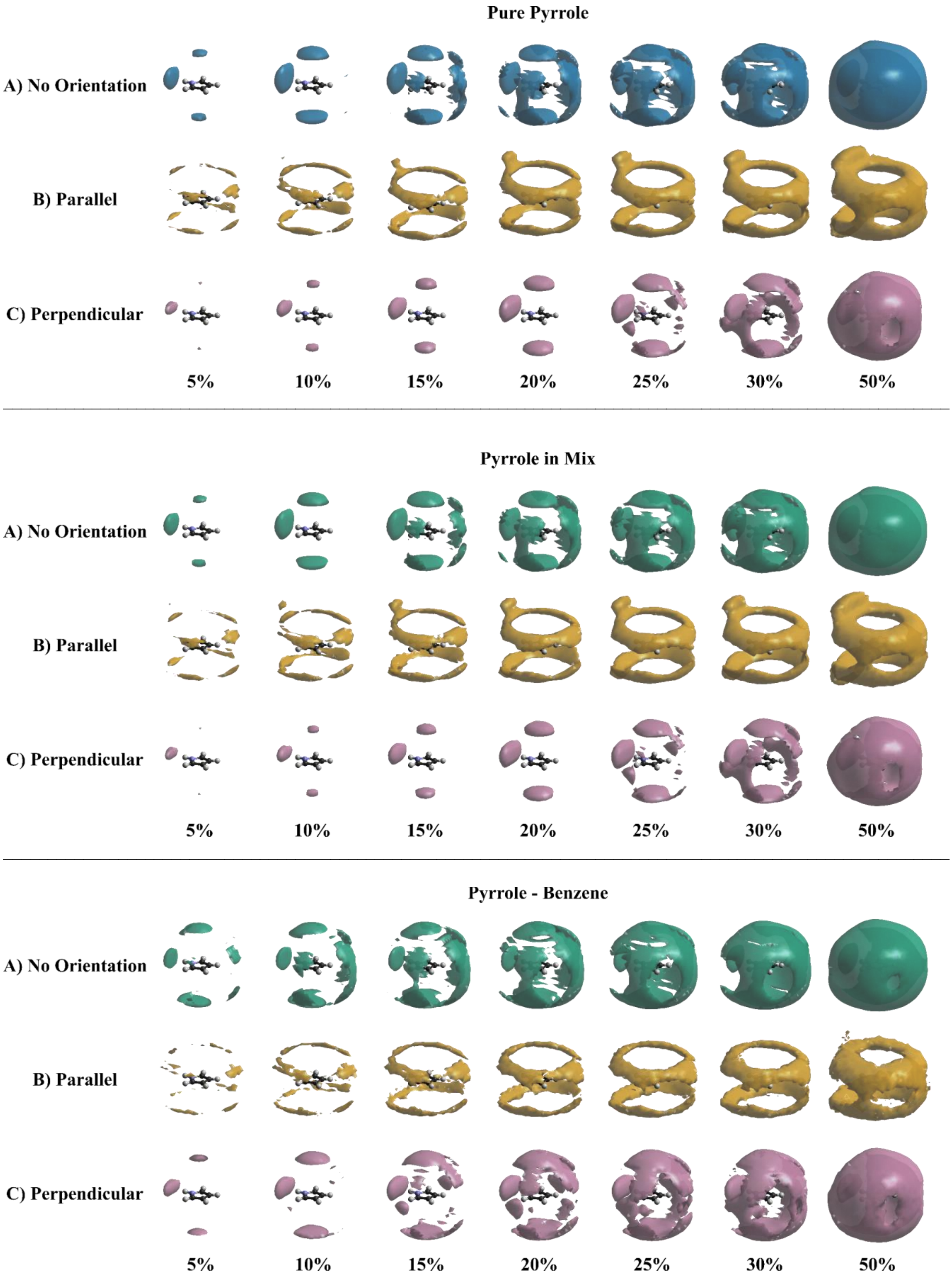
Pure Pyrrole
A) No Orientation
B) Parallel
C) Perpendicular
5%
10%
15%
20%
25%
30%
50%
Pyrrole in Mix
A) No Orientation
B) Parallel
C) Perpendicular
5%
10%
15%
20%
25%
30%
50%
Pyrrole - Benzene
A) No Orientation
B) Parallel
C) Perpendicular
5%
10%
15%
20%
25%
30%
50%

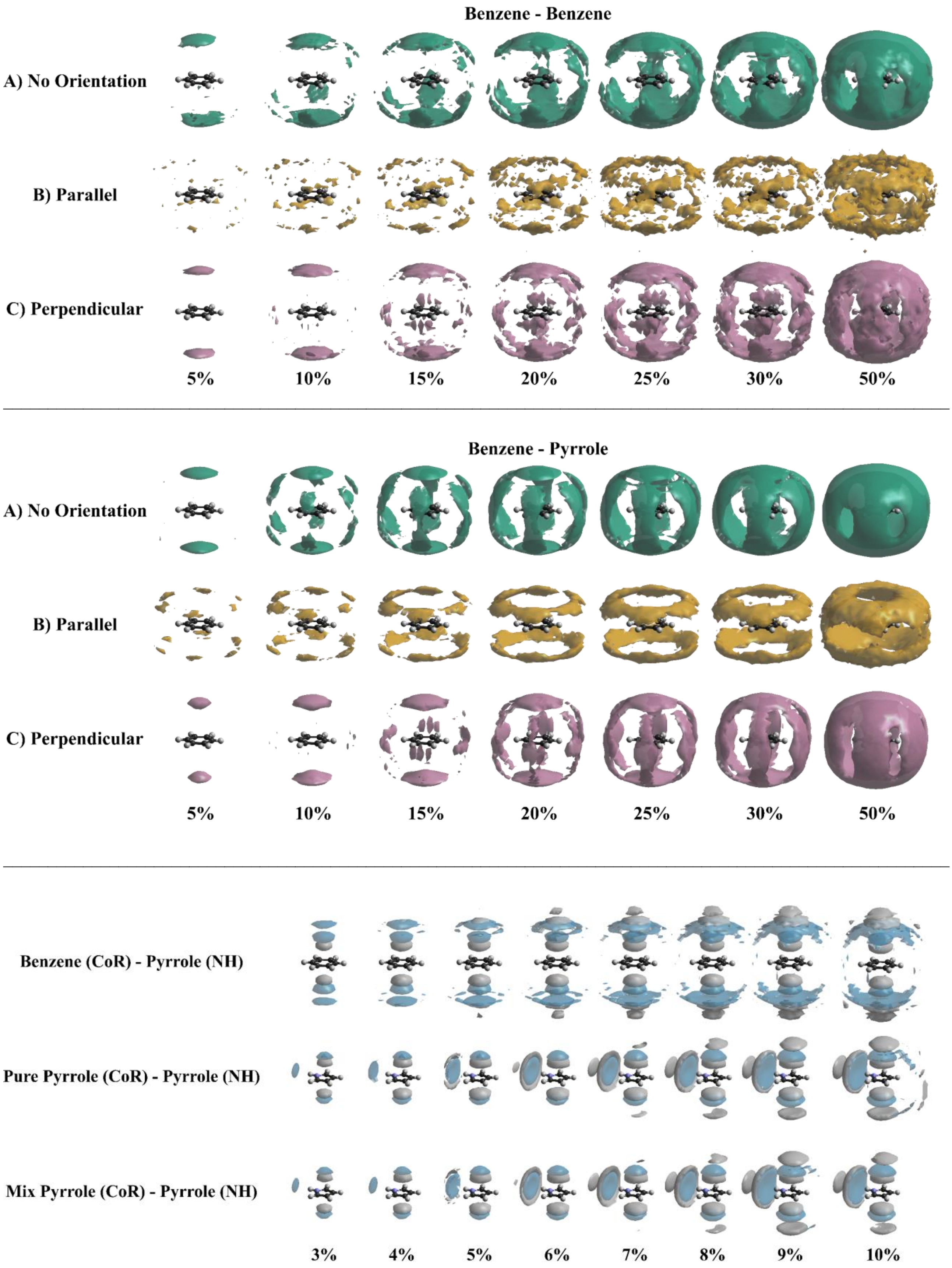

Supplementary Figure 9. *Spatial Distribution Functions, SDFs, at different percentages of visualisation.*

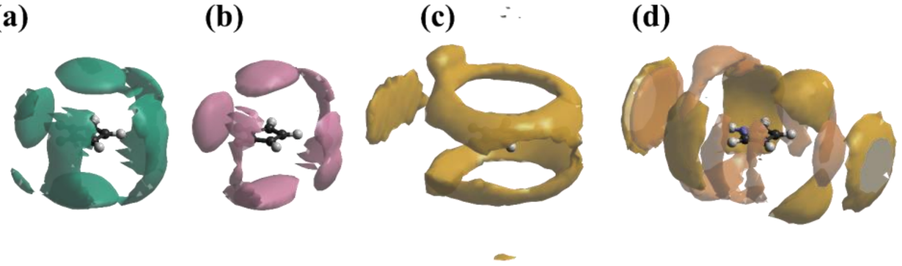

Supplementary Figure 10. *Spatial Density Functions SDFs of pure pyrrole integrated up to 12 Å.* ***(a)*** *Random orientation;* ***(b)*** *Perpendicular z∠z=90±10°;* ***(c)*** *Parallel z∠z=0±10°;* ***(d)*** *Parallel (yellow) x∠x=0±10° and antiparallel (orange) x∠x=180±10°.*

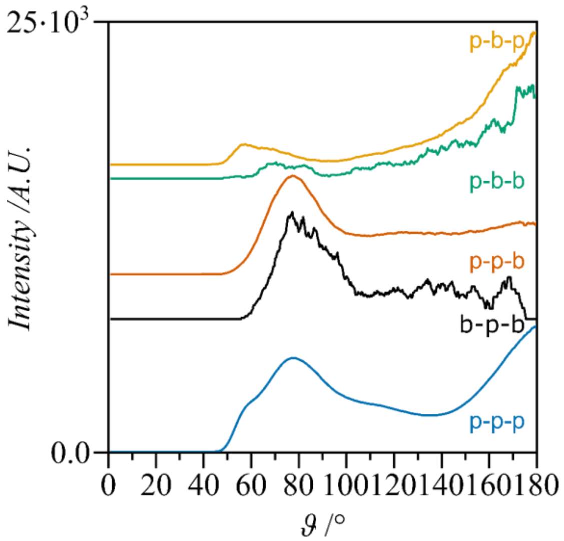

Supplementary Figure 11 *Angular distribution of all possible pyrrole-containing triplets in which a central aromatic (benzene* ***b****, pyrrole* ***p****) is found within a cut-off distance of 4.7 Å (first minimum of CoG-CoG pyrrole-pyrrole RDF) corrected by the solid angle sinϑ.*

Supplementary Note 4: Neutron Scattering Weights

Pure Pyrrole

D_5	C	H	N
C	7.0756	17.74486	4.98484
H	17.74486	11.12556	6.250727
N	4.98484	6.250727	0.877969

H_5	C	H	N
C	7.0756	9.9484	4.98484
H	9.9484	3.4969	3.50438
N	4.98484	3.50438	0.877969

$(HD)_5$	C	H	N
C	7.0756	3.89823	4.98484
H	3.89823	0.536923	1.373174
N	4.98484	1.373174	0.877969

Benzene in Pyrrole 1:19

D_6 in D_5	C	H	N
C	10.8706	18.66775	4.932566
H	18.66775	8.014394	4.235275
N	4.932566	4.235275	0.559542

$(HD)_6$ in D_5	C	H	N
C	10.8706	17.8024	4.932566
H	17.8024	7.288593	4.038946
N	4.932566	4.038946	0.559542

D_6 in $(HD)_5$	C	H	N
C	10.8706	4.966326	4.932566
H	4.966326	0.567227	1.126743
N	4.932566	1.126743	0.559542

$(HD)_6$ in $(HD)_5$	C	H	N
C	10.8706	4.100973	4.932566
H	4.100973	0.386777	0.930414
N	4.932566	0.930414	0.559542

H_6 in D_5	C	H	N
C	10.8706	16.93705	4.932566
H	16.93705	6.597235	3.842618
N	4.932566	3.842618	0.559542

D_6 in H_5	C	H	N
C	10.8706	3.706912	4.932566
H	8.7351	1.754779	1.981789
N	4.932566	1.981789	0.559542

H_6 in H_5	C	H	N
C	10.8706	10.46581	4.932566
H	10.46581	2.519022	2.374446
N	4.932566	2.374446	0.559542

Supplementary References

1. Sears, V.F., Neutron scattering lengths and cross sections. Neutron News, **1992**, *3*, 26-37.
2. Youngs, T., dlputils; GitHub. **2024**, https://github.com/trisyoungs/dlputils.
3. Youngs, T., Dissolve: next generation software for the interrogation of total scattering data by empirical potential generation. *Mol. Phys.* **2019**, *117*, 3464-3477.
4. Gray, C.G., et al., Introduction, in Theory of Molecular Fluids: I: Fundamentals. **1984**, Oxford University Press.
5. Svishchev, I.M. and P.G. Kusalik, Structure in liquid water: A study of spatial distribution functions. *J. Chem. Phys.* **1993**, *99*, 3049-3058.
6. Jorgensen, W.L., D.S. Maxwell, and J. Tirado-Rives, Development and Testing of the OPLS All-Atom Force Field on Conformational Energetics and Properties of Organic Liquids. *J. Am. Chem. Soc.* **1996**, *118,* 11225-11236.
7. McDonald, N.A. and W.L. Jorgensen, Development of an All-Atom Force Field for Heterocycles. Properties of Liquid Pyrrole, Furan, Diazoles, and Oxazoles. *J. Phys. Chem.* B, **1998**, *102,* 8049-8059.
8. Headen, T.F., Temperature dependent structural changes in liquid benzene studied using neutron diffraction. *Mol. Phys.* **2019**, *117,* 3329-3336.
