# Supplementary material for: Cooperation-Enhanced N–H···π Hydrogen Bonds: Liquid Pyrrole and Its Mixture with Benzene
Source: J Phys Chem Lett. 2026 Feb 16;17(8):2264–70. doi: 10.1021/acs.jpclett.5c04110 (PMC12951553; doi:10.1021/acs.jpclett.5c04110)
Supplement: Supplementary file 2 [file jz5c04110_si_003.pdf]

jz-2025-041106.R1

Name: Peer Review Information for "Cooperation Enhanced N-H···π Hydrogen Bonds: Liquid Pyrrole and Its Mixture with Benzene"

First Round of Reviewer Comments

Reviewer: 1

Comments to the Author

The authors present the liquid structures of pure pyrrole and a mixture containing a tiny amount of benzene. Various intermolecular interactions, such as T-shape NH...pi and Y-shape CH...pi, are investigated by a combination of the neutron total scattering and simulation-based refinement. The extracted results of RDF, SDF, and ARDF significantly reveal the highly structured NH...pi solvation in the liquid phase. Notably, the authors successfully identified a third pyrrole molecule that serves as a cooperative effect on a strong hydrogen bond, as shown in Figure 4dii. This study shed light on the fascinating nature of liquid structures supported by reliable experimental results and precise analysis. The reviewer recommends publication of this manuscript in The Journal of Physical Chemistry Letters. However, it would be beneficial if the authors addressed the comments below.

• Abstract is too long. According to the author guidelines, an abstract of no more than 150 words should be included. The manuscript comprises approximately 220 words for the abstract. This violates the submission guidelines.

• The authors mention “This highly structured solvation behavior is dominated by cooperative cyclic motifs initiated by strong NH...pi, as...., and is also reminiscent of what has been observed in the gas phase pyrrole trimers” on pages 8-9. In fact, the gas-phase trimer adopts a closed triangular structure, with the z-z angle of 60°. The reviewer wonders whether the study of neutron-scattering experiments demonstrates a triangular structure in pure pyrrole liquid. Can we find the triangular behavior in Figure 4?

• What is the basis for the mixing ratio of pyrrole and benzene as 19:1? Is the 10:1 ratio not available?

• In the caption of Figure 3(b), we see “pyrrole/benzene 1:19”, which contradicts the description of “benzene: pyrrole, 1:19” at the second line from the bottom of page 5.

• On line 4 of page 8, the authors write “Supplementary Figure 7d”, The reviewer believes that this would be Figure 8d.

Reviewer: 2

Comments to the Author

1. What is the major advance reported in the paper?

The manuscript reports on neutron scattering analysis of the structure of pyrrole-benzene mixtures. The objective is to assess the extent of hydrogen bonding between the amine in pyrrole to the benzene ring. The authors report that the analysis suggests a similar bond length for hydrogen bonding of pyrrole with itself to pyrrole-benzene; however, the abundance (i.e., coordination number) of these structures is suppressed by approximately a factor of 2. The manuscript also discussed the 3D fluid structures between these molecular types by spatial density function presentation of the refined simulation trajectories.

2. What is the immediate significance of this advance?

As presented, the significance of the finding is unclear. It is not obvious whether existing classical forcefields and simulations can represent the approximately the same conclusions. A comparison of the reference simulation to the refined results following EPSR analysis was not presented. The manuscript presents a large swath of analysis of the EPSR refined simulation trajectories, but I am not convinced that the experimental design of a low concentration of benzene in pyrrole has sufficient signal to noise to clearly extract such precise conclusions.

3. Technical suggestions

1:19 seems too low of a ratio for accurate assessment of the fluid structure. While the fits are reasonable with EPSR, the variation in the samples (eg, d-Benzene + d-pyrrole vs. h-benzene + h-pyrrole) are expected to be dominated by the differences in the scattering lengths and the bonded structures. For a stronger justification of the appropriateness of a 5% molar benzene mixture in pyrrole being sufficient, it would be best to isolate the non-bonded contributions to the observed F(q) signals by subtracting the bonded scattering obtained from the known/assumed molecular structure. With this comparison, a clear assessment can be made for the various deuteration schemes to see, for example, how much the pure d-pyrrole non-bonded F(q) differs from the d-pyrrole with varying deuterations of benzene. Is there a clear trend in the results where each mixture shows trends over a q-region or does the experimental uncertainty cause variations?

I find the evidence for the conclusions to be relatively weak and reliant only upon the neutron results without context to how much the neutron results inform the results. What is missing is the extent of refinement from the reference forcefield to the final neutron conclusions. To what extent are the qualitative trends of the NH...\pi in pyrrole-pyrrole vs. pyrrole-benzene structures present in the reference simulations? Is hydrogen bonding the only possible justification of a given structure? Fluids interact through complex entropic and energetic processes and neutrons only see the structure, not the forces or "interactions". If the reference simulations reproduce the general trends/conclusions, would it be reasonable to assess the nature of the observed structures by performing a series of simulations at different temperatures where energetically driven processes should be expected to decrease in amplitude?

The SI discussed 500 benzene and 4500 pyrrole molecules for the EPSR simulation, which is not the same as the 1:19 ratio, rather is. Is this a simply typographical error or a methodological one? 500:9500 would represent the correct 1:19 ratio.

Is the benzene-pyrrole an ideal mixture for volumetric expansion as assumed in the estimation of the necessary molecular density for the EPSR analysis?

Could you add in additional discussion on the expected uncertainty associated with the elucidation of the fluid structures containing benzene? The low particle count would suggest that exceedingly long simulations would be necessary for accurate assessment of

the benzene-benzene structures in the mixture from only the finite sampling of the simulation trajectories.

The SI image files are of low resolution and difficult to read the labels, particularly Figure S2. Please consider a vector-based image format or the inclusion of tabulated data files as supplements.

This may be pedantic, but I don't see how the center of ring and center of gravity coincide for pyrrole. NH has a molar mass of 15 while CH is 13. Even if weighted atom instead of mass, the NH bond differs from CH bond length, pulling the CoG away from the CoR. Perhaps a difference below the resolution of the imagery but it is not strictly true. This is stated at least in the Fig. 2 caption (CoG and CoR coincide for these "systems").

Ratios of 1:19 and 19:1 are both reported in the manuscript. Please use a consistent convention for clarity. CoG and CoR usage may appear in the manuscript before formal definition.

D-pyrrole synthesis and use: Is neutron scattering amplitude sufficient to estimate the percentage deuteration? Was NMR performed on the synthesized material to confirm deuteration and site replacement? Further, could the authors clarify if the 50:50 blend was produced prior to the knowledge of the 86% deuteration if that number was obtained by the neutron experiments?

Author's Response to Peer Review Comments:

DEPARTMENT OF CHEMISTRY
Physical and Theoretical Chemistry Laboratory
South Parks Road Oxford, OX1 3QZ camilla.dimino@chem.ox.ac.uk

Dr Camilla Di Mino
Glasstone Research Fellow in Chemistry
Junior Research Fellow in The Queen's College

31st January 2026

Dear Editor,

We are pleased to resubmit our manuscript "Cooperation Enhanced N-H···π Hydrogen Bonds: Liquid Pyrrole and Its Mixture with Benzene" for publication in *The Journal of Physical Chemistry Letters'* special issue: *Future Leaders in Physical Chemistry*.

We are very grateful to the referees for their time, positive comments, and recommendations, which have significantly improved the quality of the manuscript. To address questions raised by both reviewers regarding the benzene concentration, we chose the 1:19 molecular ratio (>6 atom%) to ensure that benzene was well solvated by pyrrole, while still providing measurable signal from solute centred correlations. Specifically, we have established that the average number of pyrrole molecules around each benzene is ~13. At a higher concentration, e.g. 1:10 as suggested by Reviewer 1, the benzene would not be fully solvated, and significant competitive benzene-benzene contacts would appear, complicating the interpretation of the structures of interest. In addition, we have shown in the Supplementary Information that there are significant differences, greater than the experimental uncertainties, between the pure liquid pyrrole and the benzene-pyrrole mixtures at this concentration.

We hope that after these modifications, both you and the referees will agree that our work is of sufficient quality for publication.

Yours sincerely,

Camilla Di Mino (on behalf of all authors)

Reviewer 1

The authors present the liquid structures of pure pyrrole and a mixture containing a tiny amount of benzene. Various intermolecular interactions, such as T-shape NH…pi and Y-shape CH…pi, are investigated by a combination of the neutron total scattering and simulation-based refinement. The extracted results of RDF, SDF, and ARDF significantly reveal the highly structured NH…pi solvation in the liquid phase. Notably, the authors successfully identified a third pyrrole molecule that serves as a cooperative effect on a strong hydrogen bond, as shown in Figure 4dii. This study shed light on the fascinating nature of liquid structures supported by reliable experimental results and precise analysis. The reviewer recommends publication of this manuscript in The Journal of Physical Chemistry Letters.

We thank the referee for their high praise and appreciation of our work.

However, it would be beneficial if the authors addressed the comments below.

1) Abstract is too long. According to the author guidelines, an abstract of no more than 150 words should be included. The manuscript comprises approximately 220 words for the abstract. This violates the submission guidelines.

We thank the referee for highlighting this issue. We have now shortened the abstract to within 150 words.

Changes to manuscript:

Page 1 lines 14-25

"Weak intermolecular interactions are central to the chemical and biological sciences, as they dictate the stability, growth, and geometry of larger assemblies. Among weak interactions, NH···π hydrogen bonds are abundant in structural biology, where amines interact with aromatic systems: liquid pyrrole is the ideal test solvent containing both motifs. We therefore combined total neutron scattering and simulation-based refinement to study pure pyrrole and its mixture with benzene. The NH···π interaction between pyrroles is remarkably directional, with the NH approaching the centre of the ring perpendicularly at 2.11Å. While the NH···π bond length is similar in pyrrole-pyrrole and pyrrole-benzene, the occurrence of the latter is suppressed by a factor of 2. This difference originates from cooperative mechanisms arising from the ability of pyrrole to donate and accept simultaneously a hydrogen bond. Our results clearly show that this traditionally weak interaction can become as short and directional as classical hydrogen bonds."

2) The authors mention "This highly structured solvation behavior is dominated by cooperative cyclic motifs initiated by strong NH…pi, as…., and is also reminiscent of what has been observed in the gas phase pyrrole trimers" on pages 8-9. In fact, the gas-phase trimer adopts a closed triangular structure, with the z-z angle of 60°. The reviewer wonders whether the study of neutron-scattering experiments demonstrates a triangular structure in pure pyrrole liquid. Can we find the triangular behavior in Figure 4?

We thank the referee for the suggestion. Yes, we do find the suggested triangular motif. We have added addional data (Supplementary Figure 11) where we plot the angular distribution of any 3 molecules within the first solvation shell (0 - 4.7Å). We see a clear shoulder in the distribution at ~60° that we attribute to the closed trimers, alongside the more templated structuring at ~80° and ~180°.

Changes to manuscript:

Added to Supplementary Information

“**Supplementary Figure 1** Angular distribution of all possible triplets in which a central aromatic is found within a cutoff distance of 4.7 Å (first minimum of CoG-CoG pyrrole-pyrrole RDF) corrected by the solid angle $\sin\vartheta$.”

Page 8, Line 20

“…and is also reminiscent of what has been observed in the gas phase pyrrole trimers, a motif preserved in the liquid (Supplementary Figure 11).”

3) What is the basis for the mixing ratio of pyrrole and benzene as 19:1? Is the 10:1 ratio not available?

We thank the referee for their comment. As the referee has noted correctly in their summary, the primary aim of this work is to study weak interactions from pyrrole molecules to themselves and to benzene. Hence, we deliberately chose to investigate a ratio (1:19) where each benzene molecule would be well solvated by pyrrole. In our work, we show that the average coordination number for benzene in pyrrole is ~13. At a higher concentration, e.g., 1:10, the benzene would not be fully solvated, leading to significant benzene-benzene contacts and a significant contribution from overlapping solvation shells and solvent-separated solute pairs.

In addition, at this concentration (> 6 atom % benzene) the neutron scattering contributions from benzene are distinctively measurable thanks to the high contrast provided by the opposite signs of H and D scattering lengths. If one also includes scattering contributions from pyrroles in the benzene’s first solvation shells, it can be seen that a majority of scattering events are directly influenced by benzene (please see neutron scattering weights in Supplementary Note 4). The data using a 1:19 ratio therefore allows us to probe, for example, subtle intermolecular NH···[illegible] hydrogen bonds and [illegible]···[illegible] stacking in absence of competing solute-solute interactions.

To highlight our rationale, we have added a short paragraph to the manuscript.

Changes to manuscript:

Page 3, Line 29

"The concentration has been carefully selected to ensure that at this level of dilution, the solvation of benzene is dominated by interactions with pyrrole while still providing a measurable contribution to the experimental neutron scattering signal (Supplementary Figure 2, Supplementary Note 4). This allows us to probe subtle intermolecular interactions, including benzene-pyrrole NH···□ hydrogen bonds and aromatic □ stacking."

4) In the caption of Figure 3(b), we see "pyrrole/benzene 1:19", which contradicts the description of "benzene: pyrrole, 1:19" at the second line from the bottom of page 5.

We thank the referee and we apologies for the mistake. We have now corrected it in the text.

Changes to manuscript:

*Figure 3 caption: "*benzene/pyrrole 1:19"

5) On line 4 of page 8, the authors write "Supplementary Figure 7d", The reviewer believes that this would be Figure 8d.

We thank the referee and we apologies for the mistake that has now been fixed in the text.

Changes to manuscript:

Page 8 line 11: "Supplementary Figure 8d"

Reviewer: 2

1. What is the major advance reported in the paper?

The manuscript reports on neutron scattering analysis of the structure of pyrrole-benzene mixtures. The objective is to assess the extent of hydrogen bonding between the amine in pyrrole to the benzene ring. The authors report that the analysis suggests a similar bond length for hydrogen bonding of pyrrole with itself to pyrrole-benzene; however, the abundance (i.e., coordination number) of these structures is suppressed by approximately a factor of 2. The manuscript also discussed the 3D fluid structures between these molecular types by spatial density function presentation of the refined simulation trajectories.

We thank the referee for the clear summary of our work

2. What is the immediate significance of this advance?

As presented, the significance of the finding is unclear. It is not obvious whether existing classical forcefields and simulations can represent the approximately the same conclusions. A comparison of the reference simulation to the refined results following EPSR analysis was not presented.

We agree with the referee that a comparison between the refined and unrefined total structure factors was not presented and may be of interest to readers. We have therefore added the unrefined modelled structure factors and relative residual functions in Supplementary Figure 1. The empirical potential contribution is significant but not overbearing, and it drove the structure factors towards better agreement with the experimental data. While classical force fields are a very powerful tool for understanding the structure of disordered materials, subtle interactions and many body effects such as cooperation are often not captured.

Changes to manuscript

Supplementary Information Page 3, line 6

"Supplementary Figure 1 Total structure factors F(Q)s of Figure 3 plotted between 0-5 Å^{-1} to highlight the extent of the refinement. Data (blue, yellow circles), *Dissolve* modelled without refinement (grey, long dash), EPSR refined (black, solid), and fit residuals."

The manuscript presents a large swath of analysis of the EPSR refined simulation trajectories, but I am not convinced that the experimental design of a low concentration of benzene in pyrrole has sufficient signal to noise to clearly extract such precise conclusions.

To establish that the scattering signal from the benzene is measurable outside the experimental uncertainty, we have plotted the unbound F(Q)s for pure pyrrole and benzenepyrrole mixtures in Supplementary Figure 2.

We selected a ratio (1:19) where each benzene molecule would be well solvated by pyrrole. In our work, we show that the average coordination number for benzene in pyrrole is ~13. At a higher concentration, e.g., 1:10, the benzene would not be fully solvated, leading to significant benzene-benzene contacts and a significant contribution from overlapping solvation shells, and solvent separated solute pairs.

In addition, at this concentration (> 6 atom % benzene) the neutron scattering contributions from benzene are clearly measurable thanks to the high contrast provided by the opposite signs of H and D scattering lengths (neutron weights in Supplementary Note 4). If one also includes scattering contributions from pyrroles in the benzene's first solvation shells, a majority of scattering events are directly influenced by benzene. The data using a 1:19 ratio therefore allows us to probe, for example, subtle intermolecular NH···□ hydrogen bonds and □···□ stacking in absence of competing solute-solute interactions.

To highlight our rationale, we have added a short paragraph to the manuscript.

Changes to manuscript:

Page 3, Line 29

"The concentration has been carefully selected to ensure that at this level of dilution, the solvation of benzene is dominated by interactions with pyrrole while still providing a measurable contribution to the experimental neutron scattering signal (Supplementary Figure 2, Supplementary Note 4). This allows us to probe subtle intermolecular interactions, including benzene-pyrrole NH···□ hydrogen bonds and aromatic □ stacking."

3. Technical suggestions

1:19 seems too low of a ratio for accurate assessment of the fluid structure. While the fits are reasonable with EPSR, the variation in the samples (eg, d-Benzene + d-pyrrole vs. h-benzene + hpyrrole) are expected to be dominated by the differences in the scattering lengths and the bonded structures. For a stronger justification of the appropriateness of a 5% molar benzene mixture in pyrrole being sufficient, it would be best to isolate the non-bonded contributions to the observed F(q) signals by subtracting the bonded scattering obtained from the known/assumed molecular structure. With this comparison, a clear assessment can be made for the various deuteration schemes to see, for example, how much the pure d-pyrrole non-bonded F(q) differs from the dpyrrole with varying deuterations of benzene. Is there a clear trend in the results where each mixture shows trends over a q-region or does the experimental uncertainty cause variations?

The neutron scattering contributions from benzene are measurable outside the experimental uncertainty as shown in Supplementary Figure 2, where we compare pure pyrrole to the benzene-pyrrole mixtures. We selected a ratio (1:19) where each benzene molecule is well solvated by pyrrole. In fact, the average coordination number is ~13. At a

higher concentration, e.g., 1:10, the benzene would not be fully solvated but significant benzenebenzene contacts would appear disrupting the solvation shells.

In addition, the bonded and non-bonded structures dominate in different Q intervals for molecular liquid systems constituted of small molecules. The bonded structure will contribute more to the total signal at high Q (>3 Å^{-1}), while at intermediate Q (0.5 – 3 Å^{-1}) intermolecular structure dominates. However, we have plotted the unbound F(Q) in the Supplementary Information (Supplementary Figure 2) where we show that the trend is the same as anticipated by the reviewer with significant and appreciable differences greater than the experimental uncertainties between the pure pyrroles and the mixtures, showing that the chosen ratio is not too low.

Changes to manuscript:

Supplementary Information Page 3, line 12

"**Supplementary Figure 2** Unbound F(Q)s obtained by subtracting the bound *F(Q)s* produced by *Dissolve* from the experimental neutron data, plotted with the relative experimental uncertainties. The contribution of the intermolecular structure is dominant in the range 0.1 – 3.0 Å^{-1}. The scattering intensities vary in a trend consistent with the scattering length densities of H and D samples."

I find the evidence for the conclusions to be relatively weak and reliant only upon the neutron results without context to how much the neutron results inform the results. What is missing is the extent of refinement from the reference forcefield to the final neutron conclusions.

To allow the differences to be interrogated, the unrefined modelled F(Q)s are now presented in Supplementary Figure 1, alongside refined model and their respective residuals.

To what extent are the qualitative trends of the NH…\pi in pyrrole-pyrrole vs. pyrrole-benzene structures present in the reference simulations? Is hydrogen bonding the only possible justification of a given structure? Fluids interact through complex entropic and energetic processes and neutrons only see the structure, not the forces or "interactions". If the reference simulations reproduce the general trends/conclusions, would it be reasonable to assess the nature of the observed structures by performing a series of simulations at different temperatures where energetically driven processes should be expected to decrease in amplitude?

Similar NH··· motifs as a function of the temperature have been seen before computationally via MD simulations and OPLS-AA force field for pure pyrrole (Gao, W et al., A theoretical study of N–H···π H-bond interaction of pyrrole: from clusters to the liquid. 2012, Mol. Phys. 110, 2151. https://doi.org/10.1080/00268976.2012.666277), as cited in our originally submitted manuscript (reference #9). We fully expect similar effects to be present in the unrefined model. The constraint of the experimental data has allowed us to capture cooperative effects that hadn't been seen previously by classical simulations.

To answer if hydrogen bonding is the only possible justification to the structures we see, we note that the EPSR gives the most disordered structure compatible with the neutron data. The structures we see are very short and directional, therefore assignment to HB is the only reasonable justification for such ordered structures. In addition, hydrogen bonding has been reported previously in both the solid and gas phases, and in theoretical work.

We also agree that experiments at different temperature and benzene/pyrrole concentration would be highly desirable. However, due to the time consuming and expensive nature of these experiments we would not be able to provide an experimental benchmark for such a wide range of conditions which we believe is essential to capture challenging solution behaviours. We excitedly note that upcoming modifications to the SANDALS diffractometer scheduled for 2027 will dramatically increase throughput, sufficient to collect the data necessary to draw meaningful trends. As such, we look forward to testing the reviewer's theory experimentally.

We have added a paragraph in the main text to clarify this point further.

Changes to manuscript:

Added Supplementary Figure 1: "Supplementary Figure 1 Total structure factors F(Q)s of Figure 3 plotted between 0-5 Å^{-1} to highlight the extent of the refinement. Data (blue, yellow circles), *Dissolve* modelled without refinement (grey, long dash), EPSR refined (black, solid), and fit residuals."

The SI discussed 500 benzene and 4500 pyrrole molecules for the EPSR simulation, which is not the same as the 1:19 ratio, rather is. Is this a simply typographical error or a methodological one? 500:9500 would represent the correct 1:19 ratio.

We apologise for the error, which was typographical. We set-up the refinement by selecting the molecular ratio, which was multiplied by a factor of 250, and the simulation box contained 250 benzene and 4750 pyrrole molecules.

Changes to the manuscript

Supplementary Information Page 2, line 19

"Benzene-pyrrole 1:19 molecular mixture was modelled in a cubic simulation box of side 83.63
Å containing 250 molecules of benzene and 4750 of pyrrole at a density of 0.960 g·cm^{-3} (0.0863 atoms·Å^{-3}), obtained from a weighted average of pyrrole and benzene densities with the relative molecular abundances that reflect accurately the experimental densities measured via Anton Paar density meter DMA 4100M (0.966±0.001 g·cm^{-3} for pure pyrrole and
0.960±0.001 g·cm^{-3} for pyrrole benzene)."

Is the benzene-pyrrole an ideal mixture for volumetric expansion as assumed in the estimation of the necessary molecular density for the EPSR analysis?

The referee is correct in not assuming a priori that these would be ideal mixtures. We therefore measured the densities with a density meter. The theoretical density of the benzene mixture we used in the simulations matches the experimental density with an accuracy within experimental uncertainty. We have added these measurements to the method section.

Changes to manuscript:

Supplementary Information page 2 lines 20-22

"Pure liquid pyrrole was modelled in a cubic simulation box of side 48.66 Å containing 1000 molecules to reproduce the experimental density of 0.966 g·cm^{-3} (0.0868 atoms·Å^{-3}). Benzenepyrrole 1:19 molecular mixture was modelled in a cubic simulation box of side 83.63 Å containing 500 250 molecules of benzene and 4500 4750 of pyrrole at a density of 0.960 g·cm ^{3} (0.0863 atoms·Å^{-3}). The density of the mixture was calculated from a weighted average of pyrrole and benzene densities with the relative molecular abundances that reflected accurately the experimental densities measured via Anton Paar Density Meter DMA 4100M
(0.966±0.001 g·cm^{-3} for pure pyrrole and 0.961±0.001 g·cm^{-3} for pyrrole benzene)."

Could you add in additional discussion on the expected uncertainty associated with the elucidation of the fluid structures containing benzene? The low particle count would suggest that exceedingly long simulations would be necessary for accurate assessment of the benzene-benzene structures in the mixture from only the finite sampling of the simulation trajectories.

We thank the referee for the suggestion. We have added a sentence in the method section in Supplementary Information.

Changes to manuscript:

Supplementary Information Page 2 lines 13-15

"The simulation subsequently accumulates >100,000 configurations from which we extract the average local and intermediate-range structure of the system. The high number of configurations was essential to accurately capture benzene-benzene contacts in solutions at this concentration, being benzene the minority species."

The SI image files are of low resolution and difficult to read the labels, particularly Figure S2. Please consider a vector-based image format or the inclusion of tabulated data files as supplements.

We have exported png files at 2000 DPI from Inkscape. While we cannot account for any compression which may occur during pdf conversion, we will make sure to provide the vector files or the images in a suitable format for the final article.

This may be pedantic, but I don't see how the center of ring and center of gravity coincide for pyrrole. NH has a molar mass of 15 while CH is 13. Even if weighted atom instead of mass, the NH bond differs from CH bond length, pulling the CoG away from the CoR. Perhaps a difference below the resolution of the imagery but it is not strictly true. This is stated at least in the Fig. 2 caption (CoG and CoR coincide for these "systems").

The referee is correct, and Centre-of-Ring and Centre-of-Gravity do not coincide. The CoG we refer to in the manuscript is not a Centre-of-Gravity but a Centre-of-Geometry, where the atom positions are averaged, but not weighted. As the NH bond length (1.01 Å) differs just slightly from the CH bond length (1.08 Å) (molecular geometries presented in Supplementary Table 4), the CoG and CoR are almost indistinguishable, but we agree that it is not exact. The comment in the caption had previously been added for clarity, but it has now been removed to prevent unnecessary confusion.

We would like to reassure the referee by confirming that in our analysis we have considered CoR and CoG separately and used the appropriate term where relevant throughout.

Changes to manuscript

Figure 1 Caption deleted

"Note that centre of geometry CoG and CoR coincide for these systems".

Ratios of 1:19 and 19:1 are both reported in the manuscript. Please use a consistent convention for clarity. CoG and CoR usage may appear in the manuscript before formal definition.

We apologies for the confusion, we have now corrected ratios and defined the abbreviation.

Changes to manuscript:

Page 5 line 19

"The solvation of pure pyrrole is constituted by two well-defined solvation shells, that extend from 4 to 12 Å, as seen from the Centre-of-Geometry (CoG) – CoG partial distribution functions $g_{CoG-CoG}(r)$ (Figure 4b)."

Figure 3 caption:

"benzene/pyrrole 1:19"

D-pyrrole synthesis and use: Is neutron scattering amplitude sufficient to estimate the percentage deuteration? Was NMR performed on the synthesized material to confirm deuteration and site replacement? Further, could the authors clarify if the 50:50 blend was produced prior to the knowledge of the 86% deuteration if that number was obtained by the neutron experiments?

Total neutron scattering data are normalised to absolute units which means that the intensities of the peaks and their integrals provide quantitative information about the isotopic composition. This effect is particularly powerful for hydrogen where the scattering length of protium is negative and the one of deuterium is positive. Resultantly, the scattering amplitude is sufficient to quantify the H:D ratio in our samples. NMR would not

provide a percentage of substitution as there is no perfectly (non-)deuterated H site to calibrate against. We have added a sentence in the main text and a table in the Supplementary Information for clarity.

The 50:50 equimolar mixture was produced before measuring the level of deuteration. We have added a comment for clarity.

Changes to manuscript:

Page 4 Line 4

" Given the normalisation to absolute units of neutron data, we were able to determined residual hydrogenation from the neutron scattering levels and total pair distribution functions peak intensities, with this level of hydrogenation taken into account in the data-refinement." Page 4 Line 6

"Partially hydrogenated/deuterated samples were obtained by making a D_5/H_5 equimolar mixture to obtain $(HD)_5$ pyrrole (Figure 3) before establishing the overall level of deuteration (sample preparation in Supplementary Table 2)."

Supplementary Table 2

"Experimental weights and molecular ratios used to produce the isotopically distinct samples."
